# A dual-mode LiDAR system enabled by mechanically tunable hybrid cascaded metasurfaces

**DOI:** 10.1038/s41377-025-01999-4

**Published:** 2025-08-25

**Authors:** Lingyun Zhang, Chi Zhang, Li Zhang, Jianing Yang, Wei Bian, Rui You, Xiaoli Jing, Fei Xing, Zheng You, Xiaoguang Zhao

**Affiliations:** 1 State Key Laboratory of Intelligent Manufacturing Equipment and Technology, School of Mechanical Science and Engineering, Huazhong University of Science and Technology, Wuhan, 430074 China; 2 Department of Precision Instrument, Tsinghua University, Beijing, 100084 China; 3 Laboratory of Intelligent Microsystems, Beijing Information Science and Technology University, Beijing, 100192 China; 4 State Key Laboratory of Precision Measurement Technology and Instrument, Tsinghua University, Beijing, 100084 China; 5 Beijing Advanced Innovation Center for Integrated Circuits, Tsinghua University, Beijing, 100084 China

**Keywords:** Metamaterials, Imaging and sensing

## Abstract

Light detection and ranging (LiDAR) is widely used for active three-dimensional (3D) perception. Beam scanning LiDAR provides high accuracy and long detection range with limited detection efficiency, while flash LiDAR can achieve high-efficiency detection through the snapshot approach at the expense of reduced accuracy and range. With the synergy of these distinct detection approaches, we develop a miniaturized dual-mode, reconfigurable beam forming device by cascading Pancharatnam-Berry phase and propagation phase metasurfaces, integrated with a micro-actuator. By modulating incident light polarization, we can switch the output beam of the device between the beam array scanning mode and flash illuminating mode. In the scanning mode, the device demonstrates a continuously tunable angular resolution and a ± 35° field of view (FoV) through driving the micro-actuator to achieve the lateral translation of ±100 μm. In the flash mode, uniform illumination across the entire FoV is achieved. As a proof of concept, we propose an adaptive 3D reconstruction scheme that leverages the device’s capability to switch operation modes and adjust detection resolution. Together, the proposed device and the detection scheme constitute a dual-mode LiDAR system, demonstrating high adaptability to diverse environments and catalyze the applications of more efficient and compact 3D detection systems.

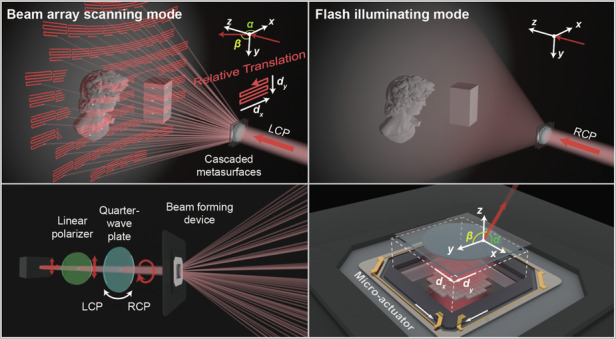

## Abstract (web summary)

Hybrid cascaded metasurfaces enable a dual-mode LiDAR system that integrates beam array scanning and flash illuminating modes for adaptive and efficient 3D sensing.

## Introduction

Light detection and ranging (LiDAR) serves as a pivotal technology in active three-dimensional (3D) imaging, providing significant advantages such as direct acquisition of depth information and strong resistance to environmental noises. These characteristics have made it indispensable in various applications, including autonomous driving^1^, smart robotics^2^, geographical mapping^3^, industrial production^4^, among others. LiDAR systems operate by emitting laser beams and processing the reflected or scattered echoes to acquire 3D information about the environment^5^. As an essential sensing device for the future intelligent society, LiDAR is rapidly advancing towards miniaturization, multifunctionality, and enhanced environmental adaptability with the aid of nanophotonic devices such as metasurfaces^6,7^. As an emerging generation of optical components, metasurfaces employ precisely designed sub-wavelength unit cells to enable unprecedented control over light’s amplitude, phase, and polarization. Metasurfaces are characterized by their planar profiles, light weight, design flexibility and compatibility with semiconductor fabrication processes^8–11^, which opens new avenues for implementing more compact and efficient beam projection/detection devices in LiDAR applications^12–14^.

The light projection method in commercial LiDAR can be categorized into two types: collimated beam scanning and wide field-of-view (FoV) flash-illumination^6^. The beam scanning LiDAR typically utilizes movable components, such as mechanically rotational mirrors/prisms or micro-electro-mechanical-systems (MEMS) micromirrors, to dynamically steer laser beams and achieves high-speed scanning across the targeted FoV^15^. Recent research has focused on the development of tunable metasurface-based systems to achieve miniaturized and high-precision beam scanning. One approach utilizes solid-state systems, where beam scanning is controlled by applying voltage to materials such as liquid crystals^16^, graphene^17^, and transparent conductive oxides^18,19^. Another approach relies on external mechanical adjustments, including movable light sources^20,21^ and MEMS actuation^14,22,23^. Despite these advancements, most studies still face limitations in terms of single-beam projection, narrow FoV, or discrete deflection angles, posing challenges in achieving practical high-performance LiDAR. On the contrary, flash LiDAR performs instantaneous 3D detection by illuminating the entire FoV at the same time, leading to significantly improved detection efficiency and frame rate. And its solid-state nature ensures impact resistance, little wear/creep, and a prolonged lifespan^24^. Metasurfaces contribute to this area by enabling high-density, static beam forming across a wide FoV through advanced techniques such as higher-order diffraction^25,26^, holographic point cloud^27^, among others. Nevertheless, the widespread illumination characteristic of flash LiDAR causes the dispersion of its limited optical power, leading to decreased detection accuracy and shorter detection distance^28^.

Considering the advantages of beam scanning and flash methods, we proposed a dual-mode LiDAR system leveraging tunable hybrid cascaded metasurfaces (THCMs). The input metasurface consists of a quadratic metasurface array realized by polarization-sensitive Pancharatnam-Berry (PB) phase (or geometric phase), while the phase profile of output metasurface is imparted by polarization-insensitive propagation phase, forming a hybrid cascading architecture. By modulating the polarization state of the incident light, we can use this device to switch between beam array scanning mode and flash illuminating mode. In the beam array scanning mode, the output metasurface is laterally translated by a shape-memory-alloy (SMA) based micro-actuator to project a scannable beam array, thereby achieving tunable angular resolution and enhanced detection efficiency compared to single-beam scanning. The beam array fully covers a FoV of ±35° under a small lateral displacement of ±100 μm. In the flash illuminating mode, the targeted objects are entirely and uniformly illuminated and detected in a single shot without the need for scanning, leveraging the high detection efficiency of flash LiDAR. For practical implementation of this device, a 3D reconstruction scheme is further proposed, where the flash illuminating mode is first utilized for initial coarse detection, followed by beam array scanning mode based on the initial results. Experimental results validate the theoretical design, demonstrating successful mode switching, a 3D reconstruction error below 1.02% and an angular resolution of 0.3°. The combination of the proposed dual-mode beam forming device and the detection scheme forms a dual-mode LiDAR system, which combines the strengths of both modes with improved adaptability in various environments and offers potential for widespread applications in drone navigation, autonomous driving, robot manipulation, consumer electronics, augmented reality, and virtual reality, etc.

## Results

### Design of the tunable hybrid cascaded metasurfaces (THCMs)

**Fig. 1:**
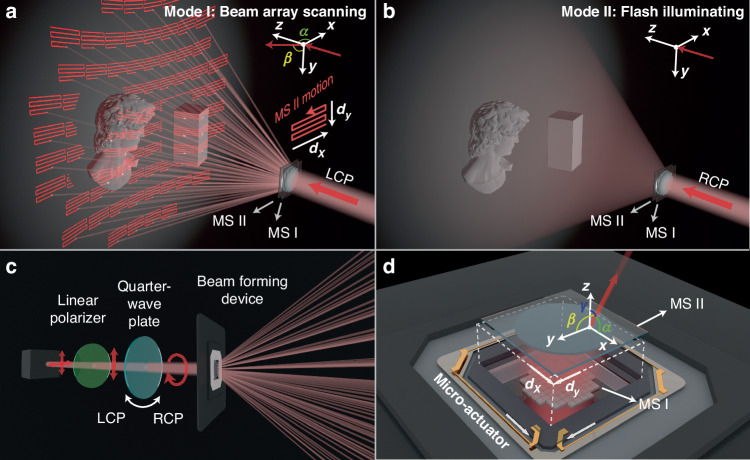
Schematic diagrams of dual-mode 3D detection using tunable hybrid cascaded metasurfaces (THCMs). **a** Schematic of beam scanning in beam array scanning mode. Left-Circularly Polarized (LCP) light passes THCMs to project a beam array. Beam scanning can be achieved by controlling the translation between the input metasurface I (MS I) and the output metasurface II (MS II). **b** Schematic of beam projection in flash illuminating mode. Right-Circularly Polarized (RCP) light passes through THCMs to achieve large-field-of-view uniform illumination. **c** Simplified optical path diagram. The beam forming device consists of the THCMs and a micro-actuator. The LCP and RCP incidence switching is achieved by controlling the angle of the Quarter-wave plate. **d** Exploded view of the beam forming device. The micro-actuator, based on shape memory alloy (SMA), controls the in-plane displacement of MS II to enable beam scanning

The overall design and operation of the dual-mode beam forming based on the THCMs, are illustrated in Fig. 1. A collimated laser beam passes through the input metasurface (MS I) and the output metasurface (MS II) sequentially. MS I and MS II are aligned parallel to the *x*-*y* plane. For left-handed circularly polarized (LCP) light incidence (Fig. 1a), the THCMs project a beam array including multiple collimated beams, each scanning across a small portion of the FoV with MS II translated in plane by *d*_*x*_ and *d*_*y*_. For right-handed circularly polarized (RCP) light incidence (Fig. 1b), the THCMs uniformly illuminate the entire FoV and target object. The polarization state of the incident light can be controlled by the relative rotation between the quarter-wave plate and the linear polarizer (Fig. 1c). This configuration may be replaced by high-efficiency LCP/RCP switchable sources that are available in recent research^29,30^. The THCMs and a customized SMA based micro-actuator (GCDS01A, GalaxyCore Inc.) are integrated together to construct the beam forming device, enabling precise translation control of MS II for beam scanning (Fig. 1d). The cooperation of the two modes of THCMs enables the flexibility and adaptability to different sensing scenarios of our proposed LiDAR system.

**Fig. 2:**
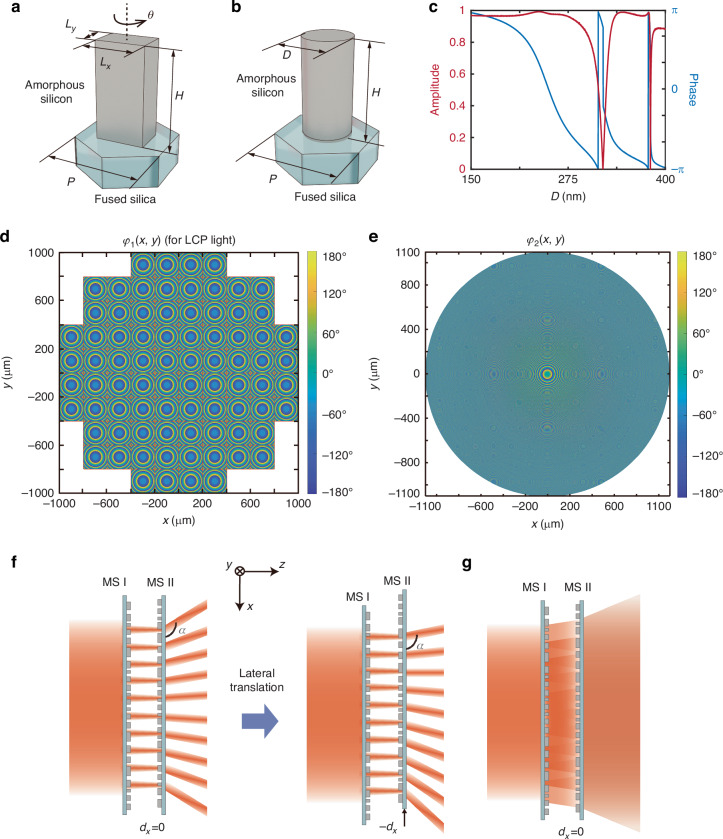
Unit cell structures and phase design of MS I and MS II, with a schematic diagram illustrating the beam array scanning mode and flash illuminating mode. **a**, **b** Schematic diagrams of the unit cell of MS I and MS II, respectively. **c** Amplitude and phase of the transmission coefficient of the unit cell of MS II under normal incidence as a function of diameter *D*. **d**, **e** Phase design of MS I and MS II, respectively. **f** Schematic diagram of beam scanning via lateral translation of MS II in beam array scanning mode. **g** Schematic diagram of achieving beam uniformity using an array design in flash illuminating mode

The two metasurfaces employ distinct phase modulation mechanisms, forming a hybrid cascading architecture that enables polarization-switchable responses. Each unit cell of the metasurfaces consists of an amorphous silicon nanopillar patterned on a fused silica substrate. MS I is a PB phase metasurface, composed of rectangular nanopillars that are identical in shape and size (Fig. 2a), but with spatially varying rotation angles *θ*. Owing to its polarization-sensitive properties, MS I can convert LCP light into RCP light and vice versa. Moreover, the phase shifts imparted to LCP and RCP light are opposite in sign (2*θ* and −2*θ*, respectively). For a near-infrared working wavelength *λ*_0_ = 1064 nm, the height *H* and period *P* are set to 600 nm and 500 nm, respectively. To maximize polarization conversion efficiency while maintaining reasonable fabrication tolerances, we set *L*_*x*_ = 295 nm and *L*_*y*_ = 165 nm as the length and width of the nanopillars in MS I, respectively (see Supplementary Note 1). MS II is a propagation metasurface, which is composed of circular nanopillars for polarization-insensitive beam shaping (Fig. 2b). By setting *H* = 600 nm and *P* = 560 nm, a full 2π phase coverage is obtained as the diameter *D* of the nanopillars in MS II varies from 150 to 400 nm, while the transmission amplitude remains close to unity (Fig. 2c).

The phase profiles of the THCMs are designed for dual-mode operations aiming at high-efficiency LiDAR system. Specifically, MS I includes a *n*_m_ × *n*_m_ periodic array of quadratic phase profile metasurfaces with a period of *P*_m_ (Fig. 2d), operating like a microlens array that divides and reforms the wavefront of the incident beam. The rotation angle *θ* distribution of unit cells in the array unit in the *i*-th row and *j*-th column in MS I can be expressed as:

1 $${\theta }_{i,j}\left(x,y\right)=\frac{p}{2}\left({\left(x-{\varGamma }_{j}\right)}^{2}+{\left(y-{\varGamma }_{i}\right)}^{2}\right)$$

where *x* ∈ [*Γ*_*j*_-*P*_m_/2, *Γ*_*j*_ + *P*_m_/2], *y* ∈ [*Γ*_*i*_-*P*_m_/2, *Γ*_*i*_ + *P*_m_/2], *p* is a real constant, and *i*, *j* = 1, 2, …, *n*_m_. The parameters (*Γ*_*j*_, *Γ*_*i*_) = ((*j-*(*n*_m_ + 1)/2)*P*_m_, (*i-*(*n*_m_ + 1)/2)*P*_m_), representing the center position of each array unit. The MS I phase profile *φ*_1*i*, *j*_(*x*, *y*) is given by 2*θ*_*i*, *j*_(*x*, *y*) for LCP light and −2*θ*_*i*, *j*_(*x*, *y*) for RCP light.

The phase profile of MS II (Fig. 2e) is:

2 $${\varphi }_{2}\left(x,y\right)=-p\left({x}^{2}+{y}^{2}\right)$$

where *x* ∈ [−(*n*_m_ + 1)*P*_m_/2, (*n*_m_ + 1)*P*_m_/2], *y* ∈ [−(*n*_m_ + 1)*P*_m_/2, (*n*_m_ + 1)*P*_m_/2]. For LCP incidence and lateral displacements *d*_*x*_ and *d*_*y*_, the total transmitted phase *φ*_*i*, *j*_(*x*, *y*) modulated sequentially by the (*i*, *j*)-th unit cell of MS I and MS II can be expressed as:

3 $$\begin{array}{ll}{\varphi }_{i,j}\left(x,y\right)={\varphi }_{1i,j}\left(x,y\right)+{\varphi }_{2}\left(x-{d}_{x},y-{d}_{y}\right)\\\qquad\qquad\; =2p\left({d}_{x}-{\varGamma }_{j}\right)x+2p\left({d}_{y}-{\varGamma }_{i}\right)y+p\left({{\varGamma }_{j}}^{2}+{{\varGamma }_{i}}^{2}-{{d}_{x}}^{2}-{{d}_{y}}^{2}\right)\end{array}$$

where *x* ∈ [*Γ*_*j*_-*P*_m_/2, *Γ*_*j*_ + *P*_m_/2], *y* ∈ [*Γ*_*i*_-*P*_m_/2, *Γ*_*i*_ + *P*_m_/2]. Based on the generalized Snell’s Law of refraction^31^ (see Supplementary Note 2), the direction angles of the output beam array for normally incident plane waves are:

4 $${\alpha }_{j}\left({d}_{x}\right)=\arccos \left(\frac{p{\lambda }_{0}}{\pi }\left({d}_{x}-{\varGamma }_{j}\right)\right)$$

5 $${\beta }_{i}\left({d}_{y}\right)=\arccos \left(\frac{p{\lambda }_{0}}{\pi }\left({d}_{y}-{\varGamma }_{i}\right)\right)$$

where the angles *α* and *β* represent the direction of the output beam relative to the *x*- and *y*-axes, respectively. Equations (4) and (5) indicate that the propagation direction of each output beam can be tuned by *d*_*x*_ and *d*_*y*_ around ${\alpha }_{j}\left(0\right)=\arccos (\frac{p{\lambda }_{0}}{\pi }{\varGamma }_{j})$ and ${\beta }_{i}\left(0\right)=\arccos (\frac{p{\lambda }_{0}}{\pi }{\varGamma }_{i})$, the entire FoV can be fully covered as long as a small *d*_*x*_ and *d*_*y*_ range of [−*P*_m_/2, *P*_m_/2] is achieved (Fig. 2f).

Additionally, the angular variation ∆*α*_*j*_ can be calculated when a small value ∆*d*_*x*_ is applied:

6 $$\Delta {\alpha }_{j}=\frac{\frac{p{\lambda }_{0}}{\pi }}{\sqrt{1-{\left(\frac{p{\lambda }_{0}}{\pi }\left({d}_{x}-{\varGamma }_{j}\right)\right)}^{2}}}{\Delta d}_{x}$$

The expression can be simplified as $\frac{p{\lambda }_{0}}{\pi }\Delta {d}_{x}$ with small *d*_*x*_ and *Γ*_*j*_ values, and ∆*β*_*i*_ holds a similar formulism. This indicates that by adjusting the translation step size, the scanning angular step can be linearly controlled, enabling the THCMs to dynamically adapt for varying environmental sensing requirements.

For the RCP incidence, the phase of MS I is reversed in sign, while the phase of MS II remains unchanged. As a result, the quadratic terms of *φ*_1*i*, *j*_(*x*, *y*) and *φ*_2_(*x*, *y*) fail to cancel each other out, leading to an overall phase profile *φ*_*i*, *j*_(*x*, *y*) possessing quadratic terms with large coefficients. In this situation, the laser beam exhibits a large divergence after passing through MS I and MS II, covering the full FoV and activating the flash illuminating mode (see Supplementary Note 3). Considering that the input beam is a Gaussian beam, the array design of MS I, compared to using an overall quadratic phase profile to achieve a wide FoV beam projection, enhances the uniformity of the output beam. The principle involves the incident beam undergoing a certain degree of divergence after passing through each array unit in MS I. As these beams further diverge through MS II, they overlap with each other, which helps to ameliorate the inhomogeneity (Fig. 2g). The uniformity of the projected light ensures the uniformity across the FoV, facilitates subsequent detection, and simplifies processing algorithms.

The design flow of the THCMs parameters is as follows. Firstly, we set the working wavelength to *λ*_0_ = 1064 nm and the beam array projection field of view (FoV) to span from −35° to +35°. Secondly, based on the discrete sampling constraint and temporarily neglecting the interlayer gap between MS I and MS II, we design the phase profile of MS II, selecting *r*_2_ = 1.1 mm and *p* = −1700 mm^−2^ to meet the FoV requirements (see Supplementary Note 4). Thirdly, considering the conditions *r*_2_ ≥ *r*_1_ + *P*_m_/2 and *r*_1_ = 0.5*n*_m_*P*_m_ for MS I, and we determine *r*_1_ = 1 mm using *P*_m_ = 200 μm and *n*_m_ = 10 by selecting the micro-actuator displacement range of ±100 μm. Fourthly, we fix the interlayer gap between MS I and MS II to 500 μm, and recalculate the quadratic phase coefficient of MS I using inverse ray tracing (see Supplementary Note 5). Finally, we perform forward ray tracing simulations to verify the system’s performance and jointly optimize the phase profiles. Keeping MS II fixed, we adjust the quadratic coefficient of MS I to minimize divergence, yielding a final value of −1319 mm^−2^, while also verifying that the THCMs meet the FoV requirement.

### Characterization of the beam forming device

**Fig. 3:**
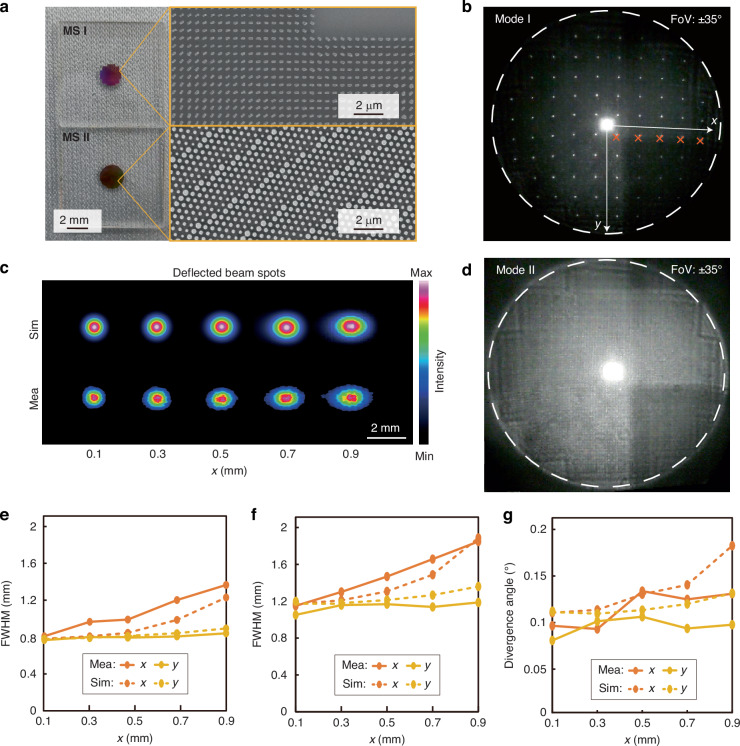
Fabricated THCMs and characterization of the dual-mode responses. **a** SEM images of the fabricated MS I and MS II. **b** Projection image of beams in beam array scanning mode. **c** Beam spot profiles for the deflected beams in beam array scanning mode. These beam spots are marked with orange Xs in (**b**). **d** Large-area illumination image in flash illuminating mode. **e**, **f** Simulated and measured FWHM values of the beam spots shown in (**b**) at distances of 10 cm and 20 cm from the emission point, respectively. **g** Simulated and measured divergence angles of the beams shown in (**b**)

To experimentally validate the proposed concept, MS I and MS II are fabricated using the EBL and ICP etching process^32^ (Fig. 3a and “Materials and Methods” section). We built a characterization system to assess the beam forming capabilities of the THCMs in both working modes (see Supplementary Note 6). Under LCP light incidence, the static beam array is shown in Fig. 3b. The intensity distributions of the output beam spots corresponding to the MS I array units, with a fixed center coordinate *Γ*_*j*_ of 0.1 mm and varying center coordinate *Γ*_*i*_ from 0.1 mm to 0.9 mm, are simulated (see Supplementary Note 7) and measured at a distance of 10 cm from MS II (Fig. 3c). Under RCP light incidence, the beam projected by THCMs covers a wide FoV, similar to flash LiDAR (Fig. 3d).

To predict the detection range, accuracy and efficiency of the LiDAR system, some principal parameters of the THCMs are evaluated under both operating modes. These parameters include the full width at half maximum (FWHM), divergence angle, angular coverage, and the energy efficiency of the output beams. In the beam array scanning mode, the FWHM in horizontal and vertical directions were measured and compared with simulation results at distances of 10 cm and 20 cm (Fig. 3e, f). Then, the divergence angle *δ* can be calculated based on the FWHM changes (Fig. 3g). The measured values exhibit good agreement with the simulated ones.

As the beam deflection angle in the horizontal direction increases, the FWHM in the horizontal direction significantly increases, and the divergence angle slightly expands. In contrast, the FWHM in the vertical direction exhibits minimal variation, with the divergence angle remaining nearly constant. The variation of the horizontal and vertical beam profiles is consistently observed in both simulation and experimental results. The simulation indicates that the difference in FWHM variation between the horizontal and vertical directions arises due to the uniform aperture of the array elements in MS I. As the horizontal deflection angle increases, the output horizontal aperture decreases, leading to enhanced diffraction effects. Conversely, the vertical aperture remains nearly unchanged, resulting in a relatively stable diffraction effect in the vertical direction. Overall, the divergence angles of the output beams remain within 0.15°, indicating high beam collimation and energy concentration. This characteristic ensures the system’s reliable detection capability within a defined operating range.

For the angular resolution, if the translation step of MS II can approach infinitesimal, the measured divergence angle *δ* can be considered equivalent to the angular resolution *Re*, in accordance with the Rayleigh criterion. When the translation step is finite, the angular resolution should take beam deflection step size into account. Assuming ∆*d*_*x*_ = ∆*d*_*y*_, the deflection step size ∆*α* can be calculated as defined in Eq. (6). Therefore, the actual resolution of the beam array scanning mode is the maximum value of these two factors, as described by the following equation:

7 $$\begin{array}{c}{Re}=\max \left(\delta ,\Delta \alpha \right)\end{array}$$

By incorporating the aforementioned system parameters, it can be deduced that for translation step sizes greater than 4.55 μm, ∆*α* is the system’s resolution, while for step sizes less than 4.55 μm, *δ* defines the resolution (see Supplementary Note 8).

By measuring the distance from MS II to the light screen and the distribution position of the beam spots, the deflection angles of the transmitted beam can be obtained, which match with the theoretical calculations well. When a micro-actuator translated MS II in the *x*-*y* plane, corresponding shifts of the beam spot positions on the screen were observed. This confirmed that when MS II translates within a two-dimensional plane by ±100 μm, the FoV of the ouput beams can dynamically cover a two-dimensional angular range of ±35° (see Supplementary Note 9). In flash illuminating mode, the coverage range of the output beam was measured to be approximated to the maximum coverage range of the beam array scanning mode. Through simulation comparisons between MS II alone and THCMs incorporating the array phase design of MS I, it was verified that the latter’s phased design is able to improve the uniformity of the output beam (see Supplementary Note 10).

Using an optical power meter, it is measured that in the beam array scanning mode, with an incident light power of 18 mW, the average output power per single-beam is 42.9 μW, resulting in an overall transmission efficiency of ~20%. In the flash illuminating mode, with same incident power, the total output power is 7.9 mW, corresponding to a transmission efficiency of 43.9%. The lower transmission efficiency observed in the beam array scanning mode is attributed to fabrication imperfections and reflection losses caused by the glass substrates, both of which can be effectively mitigated through process optimization (see Supplementary Note 11).

### 3D sensing using the dual-mode LiDAR system

**Fig. 4:**
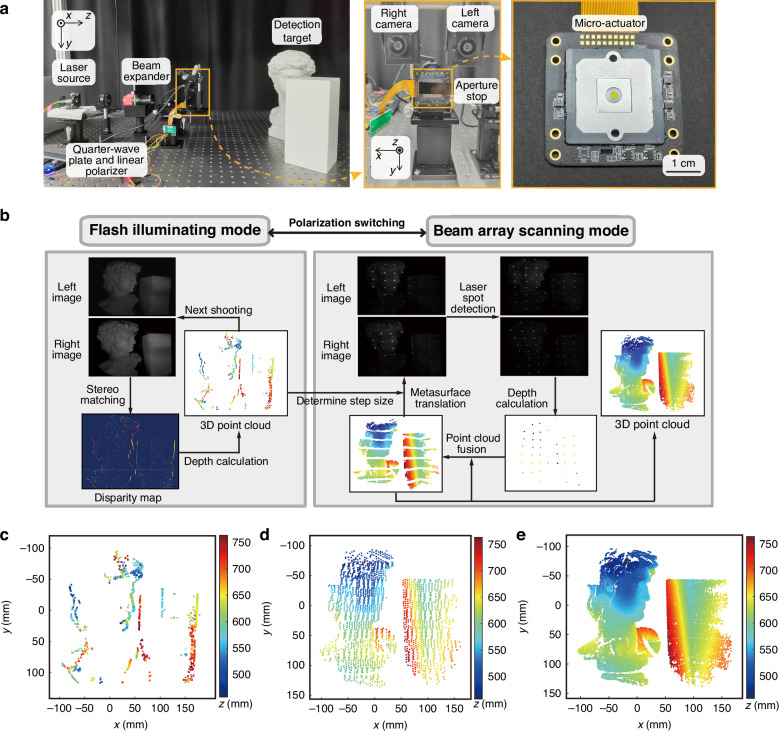
3D sensing based on the proposed LiDAR system. **a** 3D detection system based on binocular vision. **b** A scheme of the 3D detection process based on the dual-mode LiDAR. **c** 3D point cloud image obtained in flash illuminating mode. **d**, **e** 3D point cloud images obtained in beam array scanning mode through coarse scanning and fine scanning, respectively

To demonstrate the potential of the beam forming device in LiDAR applications, we have constructed a dual-mode LiDAR system (Fig. 4a) based on the principle of binocular vision, allowing for the verification of its performance in 3D imaging. A collimated laser beam passes through a linear polarizer and a rotatable quarter-wave plate for the generation of either LCP or RCP light. More compact systems can be realized by employing liquid crystal or electro-optic materials to achieve polarization state control without mechanical movement and with sub-millisecond response times. The detection of depth information is implemented using binocular vision, with two cameras co-axial in the *x* direction and at the same height in the *y* direction. By calculating and analyzing the parallax information, the depth of the detection target can be estimated.

The adaptive detection scheme of this LiDAR system combines the inherent advantages of tunable angular resolution and mode switching (see Fig. 4b and Supplementary Note 12). The flash illuminating mode facilitates instantaneous large-area sensing with high detection efficiency but offers limited resolution, accuracy, and distance. Based on the active vision, this mode is suitable for coarse 3D reconstruction and obtaining the complexity of the target of interest. The beam array scanning mode enhances the detection of detailed features of targets and enables the selection of translation steps. This capability facilitates tunable angular resolution tailored to specific targets of interest, thereby increasing the flexibility in adapting to different scenarios. However, the primary limitation of the mode is its inability to quickly obtain target features, necessitating a trade-off between detection resolution and efficiency. This makes the system’s mode switching capability essential.

The adaptive detection scheme works as follows. When detecting targets for the first time, the flash illuminating mode can be employed for snapshot sensing to capture the target’s morphology in a short period of time. Specifically, images are captured by both the left and right cameras, followed by stereo matching to compute the disparity map, which primarily captures the target’s edge information. The depth map is subsequently generated using the triangulation method. The translation step can be determined according to the obtained rough information and complexity of target objects for subsequent scans. Then, by switching the polarization state of the incident light from RCP to LCP, the system is turned to beam array scanning mode. In this mode, we capture images using the cameras, identify beam spots, and perform stereo matching to calculate the disparity of each point, determining its depth using triangulation method. Finally, the system goes back to the flash illuminating mode for next frame.

The detection results from flash illuminating mode reveal that the rough edge features of the two plaster objects were extracted (Fig. 4c), with low precision and significant noise. Subsequently, the incident light is switched to LCP light to enable the beam array scanning mode. When the rectangular plaster object is selected as the target of interest, the results from the flash illuminating mode indicate that only a coarse scan is necessary. Accordingly, the translation steps in both *x* and *y* directions are set to 50 μm, resulting in a low-resolution 3D point cloud (Fig. 4d). In contrast, when the David plaster statue is selected, the flash illuminating mode results suggest the need for a finer scan. The step size is reduced to 10 μm, yielding a high-precision 3D point cloud (Fig. 4e), with a depth error of less than 1.02% and an angular resolution of approximately 0.3°, as discussed in Supplementary Note 13. Furthermore, Supplementary Note 14 presents a comparative analysis of the proposed beam array scanning approach with existing metasurface-based beam forming methods for high-precision 3D detection. Given the required displacement range to cover the entire FoV and the experimentally characterized scanning time of 18.2 ms per 200 μm, the SMA actuator achieves a scanning frequency sufficient to support dynamic obstacle detection (see Supplementary Note 15). The proposed design demonstrates superior performance in terms of resolution, FoV, and flexibility. Together, these experiments highlight the system’s dual-mode operation capability and exceptional detection performance.

## Discussion

In this paper, we have proposed a dual-mode LiDAR system based on the THCMs integrated with a micro-actuator. By cascading PB phase metasurface and propagation phase metasurface, flexible switching between the beam array scanning mode and flash illuminating mode has been achieved. In the beam array scanning mode, both theoretical predictions and experimental results demonstrate a wide coverage of a ± 35° 2D FoV with a tunable angular resolution within a 2D translation range of ±100 μm. The divergence angles of the output beams are less than 0.15°. In the flash illuminating mode, by leveraging the array phase design, we have achieved a relatively uniform illumination across a wide FoV. Based on this integrated device, we have established an adaptive scheme to realize a dual-mode LiDAR system that is utilized for the detection of typical plaster objects. Initially, the flash illuminating mode is employed for snapshot 3D detection, capturing the edge morphology of the target and determining the translation step according to the complexity of the target. In beam array scanning mode, coarse or fine scanning is performed depending on the specific object of interest. For fine scanning, the angular resolution for constructing the 3D point cloud is approximately 0.3° at a translation step size of 10 μm, and the relative depth error for the David plaster statue is less than 1.02%.

Further efforts are expected to develop a miniaturized, high-performance LiDAR system based on the THCMs. We can incorporate compact laser sources, such as an addressable array of vertical-cavity surface-emitting lasers, to achieve highly customized detection while ensuring the emission of high-power beams to ensure sufficient detection range. Moreover, the meta-lens array has been used as a multi-functional device, serving as the beam array shaping element for structured light and the imaging component for light-field camera^33^, demonstrating its potential for the metasurface-based miniaturized depth-sensing system. These developments, combined with the versatility of our THCMs, may pave the pathway for highly adaptable miniaturized LiDAR systems that can be applied across diverse scenarios.

## Methods

### Simulation of metasurfaces

The transmission coefficients and EM field distributions of the metasurface unit cell are calculated using the CST Studio Suite 2023. Periodic boundary conditions are applied in the *x* and *y* directions to model an infinite periodic array of unit cells in the full-wave simulation. In the *z* direction, the array is surrounded by air, with perfectly matched layers at both the input and output interfaces. To meet the modulation requirements for high transmission efficiency at the near-infrared wavelength commonly used in LiDAR systems, amorphous silicon and fused silica—both nearly transparent in the near-infrared spectrum—are used as lossless materials with refractive indices of 1.4496 and 3.386, respectively.

### Fabrication of metasurfaces

The fabrication process of the device involves several steps of standard micro/nanofabrication methods (see Supplementary Note 16). First, a 500 μm thick, 4-inch fused silica glass wafer is laser-cut into smaller square pieces measuring 10 × 10 mm^2^. Then, a 600 nm thick amorphous silicon layer is deposited using plasma-enhanced chemical vapor deposition, and its thickness and dispersion properties are characterized using the ellipsometry. Afterward, a layer of positive photoresist (ZEP520A) is spin-coated onto the surface, followed by electron-beam lithography (EBL) and developing to generate the designed pattern onto the photoresist. A 50 nm chromium layer is then deposited via electron-beam evaporation, and a lift-off process is performed to create a patterned chromium mask, which serves as a hard mask for subsequent etching. Finally, inductively coupled plasma (ICP) etching is used to etch through the amorphous silicon layer, and the chromium mask is removed. MS I and MS II are fabricated separately, and MS II is integrated with a customized micro-actuator (GCDS01A, GalaxyCore Inc.) using a UV adhesive.

### Experimental characterization of the THCMs

Using the 1064 nm laser (CoLID-I-1064, Connet Laser Technology) and the fiber-coupled collimator (GCXLF11APC-1064, Daheng Optics), collimated laser beam is emitted, and the laser power can be adjusted accordingly. The deflection angles of the output beams are calculated based on the coordinates of the deflected beam spots, which are captured on a light screen. A near-infrared CCD camera is employed to identify the location of the output beam spots. A beam profiler (BP209IR1/M, Thorlabs) is installed on a motorized slide rail, which can be manually adjusted on the optical stage to align with the direction of light propagation (see Supplementary Note 6). The transmission efficiency of the THCMs is measured using a GCI-08 optical power meter (Daheng Optics).

### 3D detection based on dual-mode LiDAR

The switching between LCP light and RCP light can be achieved by rotating and adjusting the angle between a linear polarizer (LBTEK Optics) and a quarter-wave plate (LBTEK Optics). In the beam array scanning mode, the translational movement of MS II is controlled by the customized micro-actuator with a resolution of 3 μm and a travel range of ±150 μm in the *x* and *y* axes (see Supplementary Note 15). Simultaneously, two near-infrared CCD cameras are used to capture images from the left and right sides, enabling 3D detection of both the David plaster model and the rectangular plaster model in dual-mode based on the stereo vision approach.

## Supplementary information

Supplementary Information

## Data Availability

The data and codes that support the findings of this study are available from the corresponding author upon reasonable request.
