# Supplementary material for: A dual-mode LiDAR system enabled by mechanically tunable hybrid cascaded metasurfaces
Source: Light Sci Appl. 2025 Aug 25;14:287. doi: 10.1038/s41377-025-01999-4 (PMC12379129; doi:10.1038/s41377-025-01999-4)
Supplement: Supplementary file 1 — Supplementary Information [file 41377_2025_1999_MOESM1_ESM.docx]

**Supplementary Information** **for**

**A Dual-Mode LiDAR System Enabled by Mechanically Tunable Hybrid Cascaded Metasurfaces**

Lingyun Zhang,^1, †^ Chi Zhang,^2, †^ Li Zhang,^2^ Jianing Yang,^2^ Wei Bian,^2^ Rui You,^3^ Xiaoli Jing,^3,^ * Fei Xing,^2, 4, 5,^ * Zheng You^2, 4, 5^  and Xiaoguang Zhao^2, 4, 5,^ *

^1^ State Key Laboratory of Intelligent Manufacturing Equipment and Technology, School of Mechanical Science and Engineering, Huazhong University of Science and Technology, Wuhan, 430074, China.

^2^ Department of Precision Instrument, Tsinghua University, Beijing, 100084, China.

^3^ Laboratory of Intelligent Microsystems, Beijing Information Science and Technology University, Beijing, 100192, China.

^4^ State Key Laboratory of Precision Measurement Technology and Instrument, Tsinghua University, Beijing, 100084, China.

^5^ Beijing Advanced Innovation Center for Integrated Circuits, Tsinghua University, Beijing 100084, China.

^†^ These authors contributed equally to this work.

*Correspondence: [jingxiaoli@bistu.edu.cn](mailto:jingxiaoli@bistu.edu.cn) (X. J.); [xingfei@mail.tsinghua.edu.cn](mailto:xingfei@mail.tsinghua.edu.cn) (F. X.); [zhaoxg@mail.tsinghua.edu.cn](mailto:zhaoxg@mail.tsinghua.edu.cn) (X. Z.)

**Supplementary Note 1****: Determination of *L_x_* and *L_y_* parameters for PB phase metasurface unit cell**


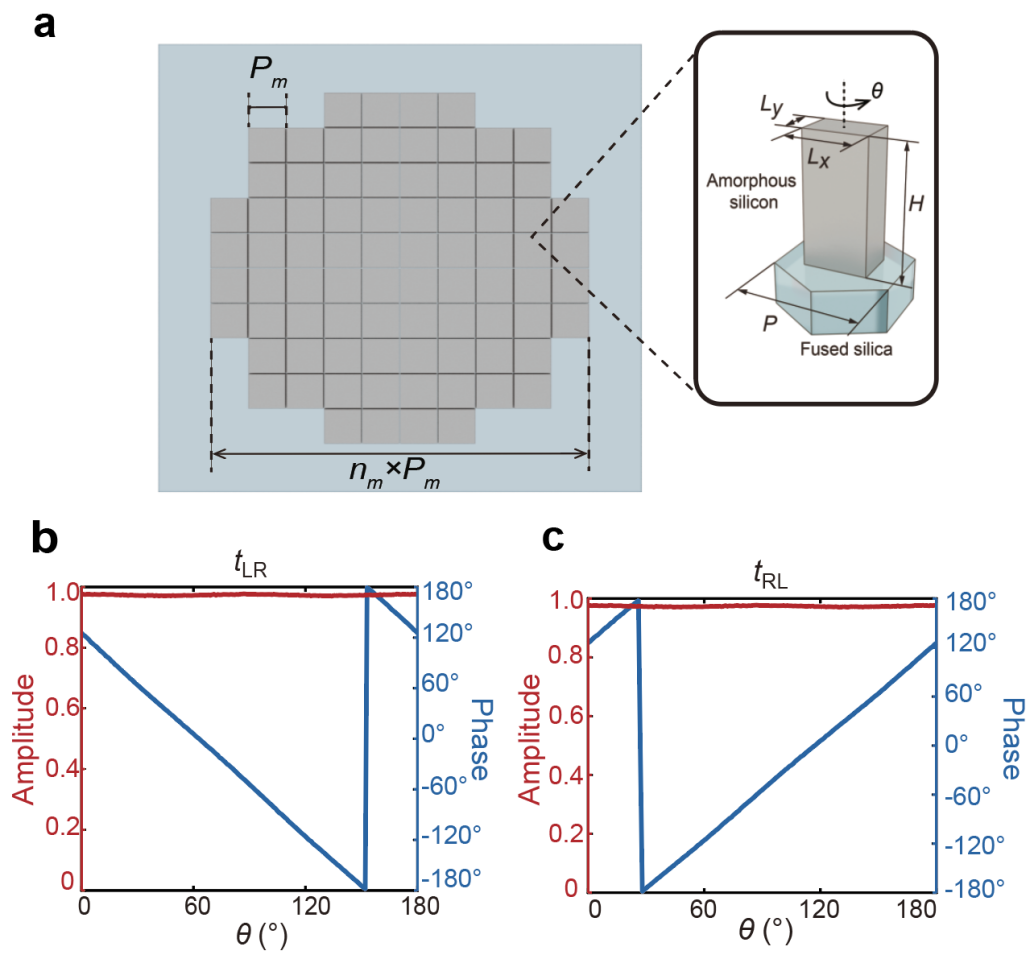


**Fig. S1 | a** Schematic diagram of MS I. **b, c** Amplitude and phase of the transmission coefficient *t*_LR_ and *t*_RL_ of the unit cell of MS I under normal incidence as functions of rotation angle *θ*.

MS I refers to the PB phase metasurface, which consists of rectangular nanopillars. The structural parameters are set as *H* = 600 nm and *P* = 500 nm. For near-infrared light with a wavelength of 1064 nm, the refractive index of amorphous silicon is 3.386, and that of fused silica is 1.4496. This unit cell enables polarization-sensitive optical field manipulation. This sensitivity is based on the near-ideal PB phase response as shown in Fig. S1. Owing to its polarization-sensitive properties, MS I can convert left-handed circularly polarized light (LCP) into right-handed circularly polarized light (RCP), and vice versa. Additionally, the phase imparted to LCP and RCP incidence is opposite.


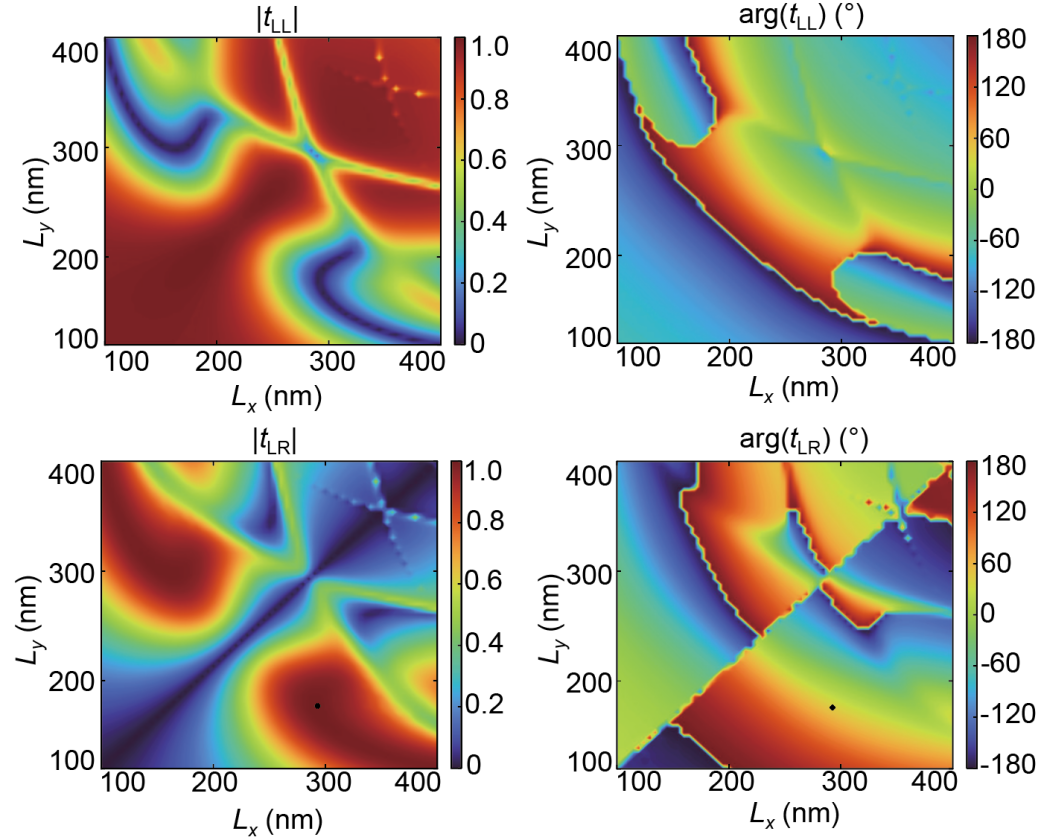


**Fig. S2 | Simulated complex transmission coefficients of the PB phase metasurface unit cell in a circularly polarized orthogonal basis at a wavelength of λ = 1064 nm.**

When the coordinate system is based on a circularly polarized orthogonal basis, the Jones matrix *J_t_*_[LR]_(*L_x_*, *L_y_*) can be used to describe the variation in the transmission coefficients:

$$\begin{aligned} \begin{aligned} J_{t\left[ \mathrm{LR} \right]}\left( L_{x},L_{y} \right)=\left[ \begin{matrix} t_{\mathrm{LL}}\left( L_{x},L_{y} \right) & t_{\mathrm{LR}}\left( L_{x},L_{y} \right) \\ t_{\mathrm{RL}}\left( L_{x},L_{y} \right) & t_{\mathrm{RR}}\left( L_{x},L_{y} \right) \end{matrix} \right] \end{aligned}\#\left( s-1 \right) \end{aligned}$$

where *t*_RR_(*L_x_*, *L_y_*)=*t*_LL_(*L_x_*, *L_y_*) and *t*_RL_(*L_x_*, *L_y_*)=*t*_LR_(*L_x_*, *L_y_*). Thus, only two coefficients are independent among *t*_LL_, *t*_LR_, *t*_RL_ and *t*_RR_. Therefore, the analysis focuses on *t*_LL_ and *t*_LR_. Using *L_x_* and *L_y_* as variables, full-wave simulations of the unit cell are performed using CST Studio Suite 2023, and the results are shown above. Specifically, select *L_x_ =* 295 nm and *L_y_ =* 165 nm, where |*t*_LR_| is maximized and the manufacturing tolerance is relatively low.

In summary, this unit cell enables polarization-sensitive optical field manipulation. This sensitivity is based on the near-ideal PB phase response. When a rotation angle *θ* is introduced, the incident LCP light is almost completely converted into an output RCP light, with a phase factor of $e^{i\left( 2\theta+\arg\left( t_{\mathrm{LR}}\left( L_{x} ,L_{y} \right) \right) \right)}$. Similarly, the incident RCP light is nearly fully converted into an output LCP light, with a phase factor of $e^{i\left( -2\theta+\arg\left( t_{\mathrm{LR}}\left( L_{x},L_{y} \right) \right) \right)}$. Additionally, the argument of arg(*t*_LR_(*L_x_*, *L_y_*)) almost fully covers the range from -π to π.

**Supplementary** **Note 2: The Derivation of Deflection Angles for Output beams**

When the spacing between MS I and MS II is neglected, under LCP light incidence, the overall phase of the cascaded metasurfaces based on Equation (3) is given by:

$$\varphi_{i,j}\left( x,y \right)=2p\left( d_{x}-\Gamma_{j} \right)x+2p\left( d_{y}-\Gamma_{i} \right)y+p\left( {\Gamma_{j}}^{2}+{\Gamma_{i}}^{2}-{d_{x}}^{2}-{d_{y}}^{2} \right)$$

where *x* ∈ [*Γ_j_*-*P*_m_/2, *Γ_j_*+*P*_m_/2], *y* ∈ [*Γ_i_*-*P*_m_/2, *Γ_i_*+*P*_m_/2]. The parameters (*Γ_j_*, *Γ_i_*) = ((*j-*(*n*_m_+1)/2*)P*_m_, (*i-*(*n*_m_+1)/2*)P*_m_), representing the center position of each array unit. Based on the Generalized Snell's Law of refraction in full space^1^, the relationship between the deflection angles of the incident and output beams, determined by the phase gradient of the metasurface, can be derived as follows:

$$\begin{aligned} \frac{\partial\varphi}{\partial x}=\frac{2\pi}{\lambda_{0}}\left( n_{t}\cos\alpha_{t}-n_{i}\cos\alpha_{i} \right)\#\left( s-2 \right) \end{aligned}$$

$$\begin{aligned} \frac{\partial\varphi}{\partial y}=\frac{2\pi}{\lambda_{0}}\left( n_{t}\cos\beta_{t}-n_{i}\cos\beta_{i} \right)\#\left( s-3 \right) \end{aligned}$$

where *α*_i_ and *β*_i_ represent the incident angles of the light, and *n*_i_ is the refractive index of the medium where the incident light is located. Similarly, *α*_t_ and *β*_t_ represent the deflection angles of the output beams, and *n*_t_ is the refractive index of the medium where the output beams are located.

In this work, both *α*_i_ and *β*_i_ are set to 90°. *n*_i_ and *n*_t_ are the refractive indices of air, which are 1. By substituting Equation (3) into Equations (S-2) and (S-3) respectively, the deflection angles of the beams can be derived as follows:

$$\begin{aligned} \alpha_{j}\left( d_{x} \right)=\arccos\left( \frac{p\lambda_{0}}{\pi}\left( d_{x}-\Gamma_{j} \right) \right), \beta_{i}\left( d_{y} \right)=\arccos\left( \frac{p\lambda_{0}}{\pi}\left( d_{y}-\Gamma_{i} \right) \right) \end{aligned}$$

**Supplementary** **Note 3: Principal analysis of dual-mode implementation in THCMs**


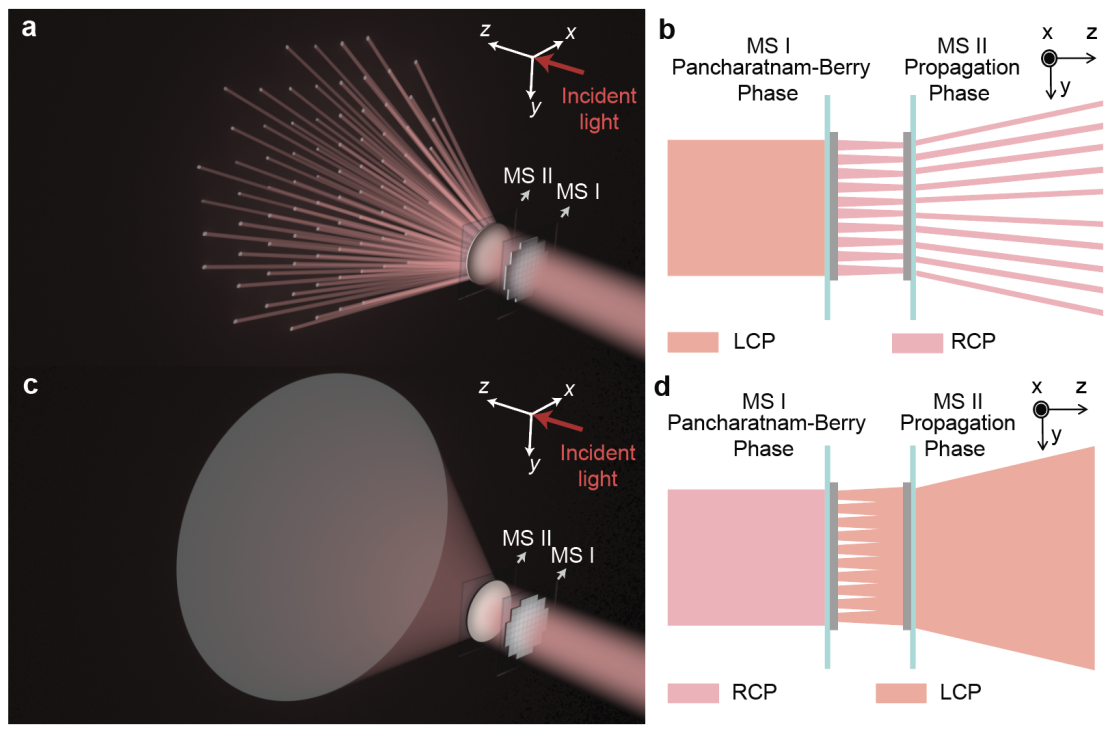


**Fig. S3 | Schematic diagram of the dual-mode implementation in THCMs. a** Beam projection diagram under the beam array scanning mode. **b** Side view of the optical path under the beam array scanning mode. **c** Beam projection diagram under the flash illumination mode. **d** Side view of the optical path under the flash illumination mode.

MS I is a PB phase metasurface, with its unit cell shown in Fig. 2a. Under LCP and RCP, the transmission equation is as follows:

$$\begin{aligned} E_{t\left[ \mathrm{LR} \right]}=J_{t\left[ \mathrm{LR} \right]}E_{\left[ \mathrm{LR} \right]}\#\left( s-4 \right) \end{aligned}$$

where *E*_[LR]_ represents the input Jones vector, *E_t_*_[LR]_ represents the output Jones vector, and *J_t_*_[LR]_ is the Jones matrix:

$$\begin{aligned} J_{t\left[ \mathrm{LR} \right]}\left( \tilde{x},\tilde{y} \right)=\left[ \begin{matrix} 0 & e^{i\left( -2\theta\left( \tilde{x},\tilde{y} \right) \right)} \\ e^{i2\theta\left( \tilde{x},\tilde{y} \right)} & 0 \end{matrix} \right]\left| t_{xx}\left( \tilde{x},\tilde{y} \right) \right|e^{i\arg\left( t_{xx}\left( \tilde{x},\tilde{y} \right) \right)}\#\left( s-5 \right) \end{aligned}$$

MS I converts incident LCP light to RCP light and incident RCP light to LCP light, while imparting opposite phases to each. The phase modulation is related to the unit cell rotation angle *θ*.

MS II is a propagation phase metasurface, with its unit cell shown in Fig. 2b. Under orthogonal polarizations in the *x* and *y* directions, the transmission equation is as follows:

$$\begin{aligned} E_{t\left[ xy \right]}=J_{t\left[ xy \right]}E_{\left[ xy \right]}\#\left( s-6 \right) \end{aligned}$$

where *E*_[_*_xy_*_]_ represents the input Jones vector, *E_t_*_[_*_xy_*_]_ represents the output Jones vector, and *J_t_*_[_*_xy_*_]_ is the Jones matrix:

$$\begin{aligned} J_{t\left[ xy \right]}\left( \tilde{x},\tilde{y} \right)=\left[ \begin{matrix} 1 & 0 \\ 0 & 1 \end{matrix} \right]\left| t_{xx}\left( \tilde{x},\tilde{y} \right) \right|e^{iarg\left( t_{xx}\left( \tilde{x},\tilde{y} \right) \right)}\#\left( s-7 \right) \end{aligned}$$

MS II is insensitive to polarization and its phase modulation is related to the unit cell diameter *D*.

When LCP light is incident, the system operates in beam array scanning mode. When the distance between MS I and MS II is set to 500 μm, the phase of MS I is *φ*_1_*_i, j_*(*x, y*) = -1319((*x*-*Γ_j_*)^2^+(*y*-*Γ_i_*)^2^). Based on the earlier explanation of reverse ray tracing, when the distance between MS I and MS II is negligible, the phase of MS I becomes *φ’*_1_*_i, j_*(*x, y*) = -1700((*x*-*Γ_j_*)^2^+(*y*-*Γ_i_*)^2^). The phase of MS II is *φ*_2_(*x, y*) = 1700(*x*^2^+*y*^2^). From the lens phase formula, it can be observed that MS I can converge the light rays, while MS II can diverge the light rays, and the focal points of the two are coincident. Thus, collimated light with a small divergence angle can be emitted, which is the basic principle of the beam array scanning mode, as shown in Fig. S3.

When RCP light is incident, the system operates in flash illumination mode. The phase of MS I becomes *φ*_1_*_i, j_*(*x, y*) = 1319((*x*-*Γ_j_*)^2^+(*y*-*Γ_i_*)^2^). The phase of MS II remains unchanged. By analogy to the principle introduced above, this will result in large-area beam illumination, similar to flash LiDAR. The incident beam undergoing a certain degree of divergence after passing through each unit in MS I. As these beams further diverge through MS II, they overlap with each other, which helps to ameliorate the inhomogeneity of output beam.

**Supplementary Note 4: Determination of the radius *r*_2_ of MS II**


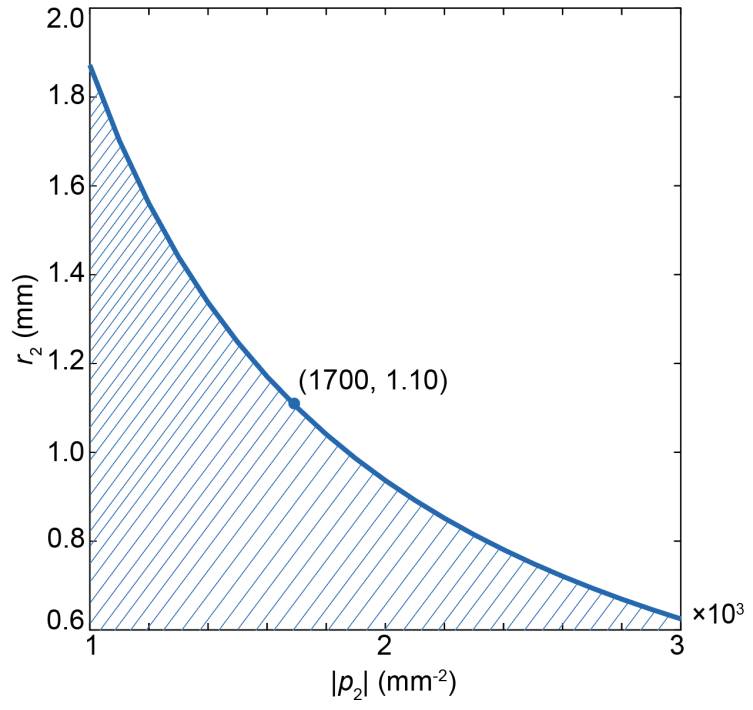


**Fig. S4 | Feasible range of values for the radius *r*_2_ of MS II.**

The phase profile of MS II is *φ*_2_(*x,y*) = *p*(*x*^2^+*y*^2^), so the magnitude of the phase gradient is:

$$\begin{aligned} \left| Grad \right|=\sqrt{\left( 2px \right)^{2}+\left( 2py \right)^{2}}=2\left| p \right|\sqrt{x^{2}+y^{2}}=2\left| p \right|r_{2}\#\left( s-8 \right) \end{aligned}$$

The minimum number of unit cells used to sample a phase variation of 2*π* at the metasurface edge can be estimated as:

$$\begin{aligned} M=\frac{\pi}{Pr_{2}\times\left| p \right|}\#\left( s-9 \right) \end{aligned}$$

where *P* is the period constant of the unit cell. If set *M* > *M*_min_, then:

$$r_{2}<r_{2\max}=\frac{\pi}{PM_{\min}\times\left| p \right|}$$

For *P* = 560 nm and *M*_min_ = 3, the feasible range of *r*_2_ is plotted as the shaded area in Fig. S4. Taking into account the actual size of the device and fabrication difficulties, set its aperture radius to 1.1 mm, *p* = 1700 mm^-2^.

**Supplementary** **Note 5: The design method of far-field coupled tunable hybrid cascaded metasurfaces (THCMs) based on reverse ray tracing technique**


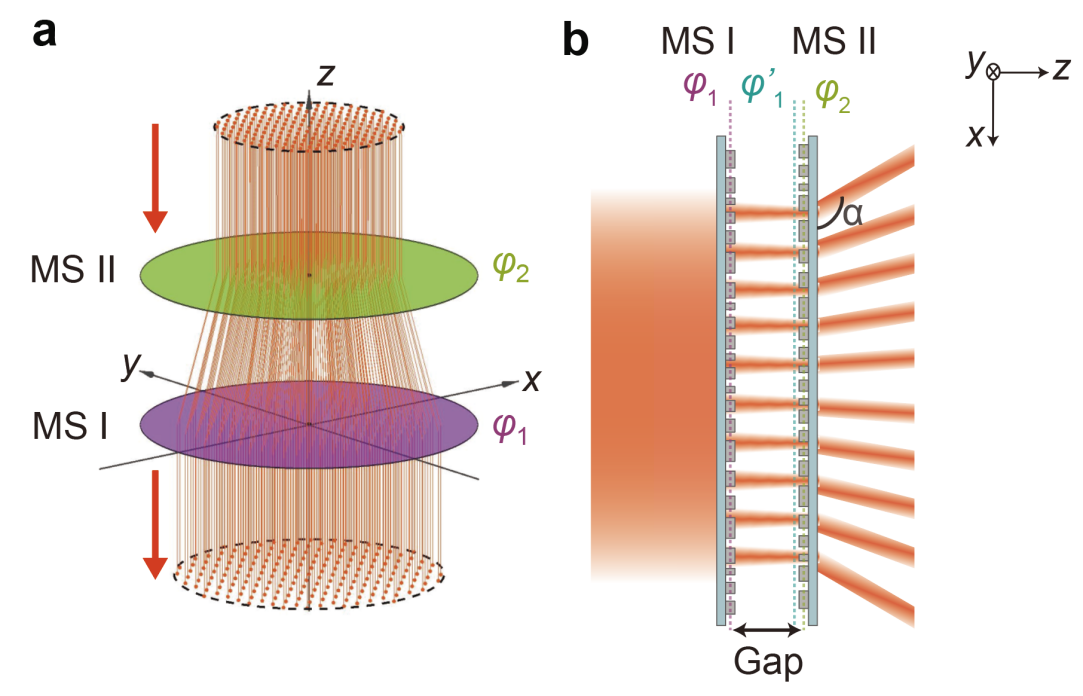


**Fig. S5 | Schematic diagram of a far-field coupled tunable hybrid cascaded metasurfaces (THCMs) using the reverse ray-tracing method. a** Illustration of a reverse ray-tracing simulation to calculate the phase profile *φ*_1_ when a constant gap separates MS I and MS II. **b** Phase profile diagram of the THCMs based on far-field coupling.

Currently, numerous studies have demonstrated tunable cascaded metasurfaces for beam manipulation. Most of these studies^2,3^ are based on near-field coupling mechanisms, where the metasurface layers are closely stacked, and the gap size is smaller than the working wavelength, leading to electric, magnetic, and evanescent field coupling. The advantage of near-field coupling lies in the fact that the overall phase of the cascaded metasurfaces can be simply represented as the direct addition of the phases of the two metasurface layers. However, such small gaps pose challenges for device integration and practical application. Therefore, this paper adopts the reverse ray-tracing method^4^ to achieve THCMs with far-field coupling.

Firstly, when the spacing between MS I and MS II is neglected, under LCP light incidence, the phase profile of MS I can be expressed as *φ*_1_*_i, j_*(*x, y*) = *p*((*x*-*Γ_j_*)^2^+(*y*-*Γ_i_*)^2^). The phase profile of MS II can be written as *φ*_2_(*x, y*) = *p*(*x*^2^+*y*^2^). Secondly, when the spacing between MS I and MS II cannot be neglected, the phase profile of MS II remains unchanged, and a new phase profile for MS I is obtained using the reverse ray-tracing method. The basic concept of reverse ray tracing is to design the phase *φ_1_* of MS I so that the distribution of light rays reaching the surface of MS II is consistent with that in the near-field coupling case.

Thus, as shown in Fig. S5, the phase profile of MS I, in the near-field coupling case, is given by: *φ’*_1_*_i, j_*(*x, y*) = -1700((*x*-*Γ_j_*)^2^+(*y*-*Γ_i_*)^2^). Through the application of the reverse ray-tracing method, the phase profile of MS I is modified to: *φ*_1_*_i, j_*(*x, y*) = -1319((*x*-*Γ_j_*)^2^+(*y*-*Γ_i_*)^2^). This adjustment ensures that the distribution of light rays reaching MS II in the far-field coupling scenario matches that of the near-field coupling case.

**Supplementary Note 6: Introduction to the testing system and procedure**


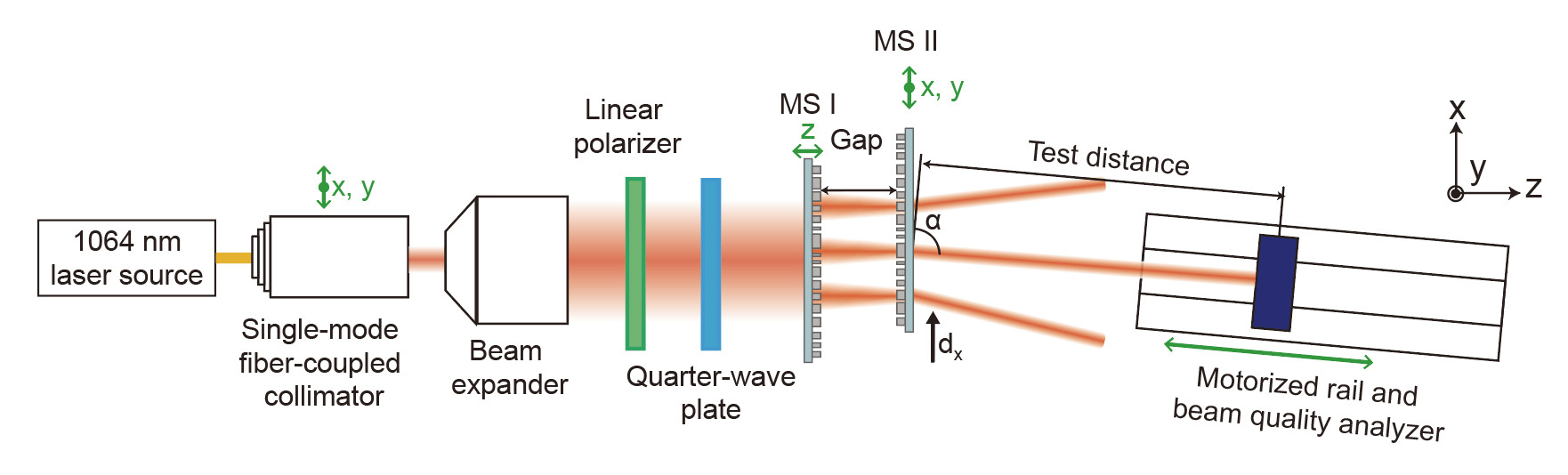


**Fig. S6 | Diagram of the experimental system for testing the beam profile of the beam forming device.**

In the beam forming device, both MS I and MS II are integrated with the shape memory alloy (SMA) micro-actuator to enable relative translation in the *x*-*y* plane. The experimental system for testing the beam profile of the beam forming device in beam array scanning mode is depicted in Fig. S6. Prior to the beam forming device, a linear polarizer and a quarter-wave plate are used to control the polarization state of the incident light. Fiber-coupled collimators are installed on pitch and yaw adjustable mirror mounts, which are mounted on a manual three-dimensional translation stage to ensure collimated beams are incident perpendicular to the center position of metasurface MS I.

After emerging from the fiber-coupled collimators, the beam passes through a beam expander to ensure uniform illumination across the metasurface. The direction angle and diameter of the output beam are measured by the light screen and beam quality analyzer, respectively. The analyzer is mounted on an electrically driven slide rail, which can be manually placed freely on the optical platform to align its track direction with the direction of beam propagation.

In the beam array scanning mode, the beam qualities are characterized by measuring the FWHM and divergence angle *δ* of the output beams. The FWHM values in both the horizontal and vertical directions are subsequently measured at distances of 10 cm and 20 cm, with the time-averaged values denoted as $\bar{D}_{x1}$, $\bar{D}_{y1}$, $\bar{D}_{x2}$ and $\bar{D}_{y2}$, respectively. The beam divergence angles *δ* are calculated based on the following equations.

$$\begin{aligned} \delta_{x}=\tan^{-1} \left| \frac{\bar{D}_{x1}-\bar{D}_{x2}}{2S} \right|\#\left( s-10 \right) \end{aligned}$$

$$\begin{aligned} \delta_{y}=\tan^{-1} \left| \frac{\bar{D}_{y1}-\bar{D}_{y2}}{2S} \right|\#\left( s-11 \right) \end{aligned}$$

where *S* is the distance difference between the two measurement locations.

**Supplementary Note 7: Optical system modeling and simulation based on Zemax OpticStudio**

***1. Simulation of dual-mode operation***


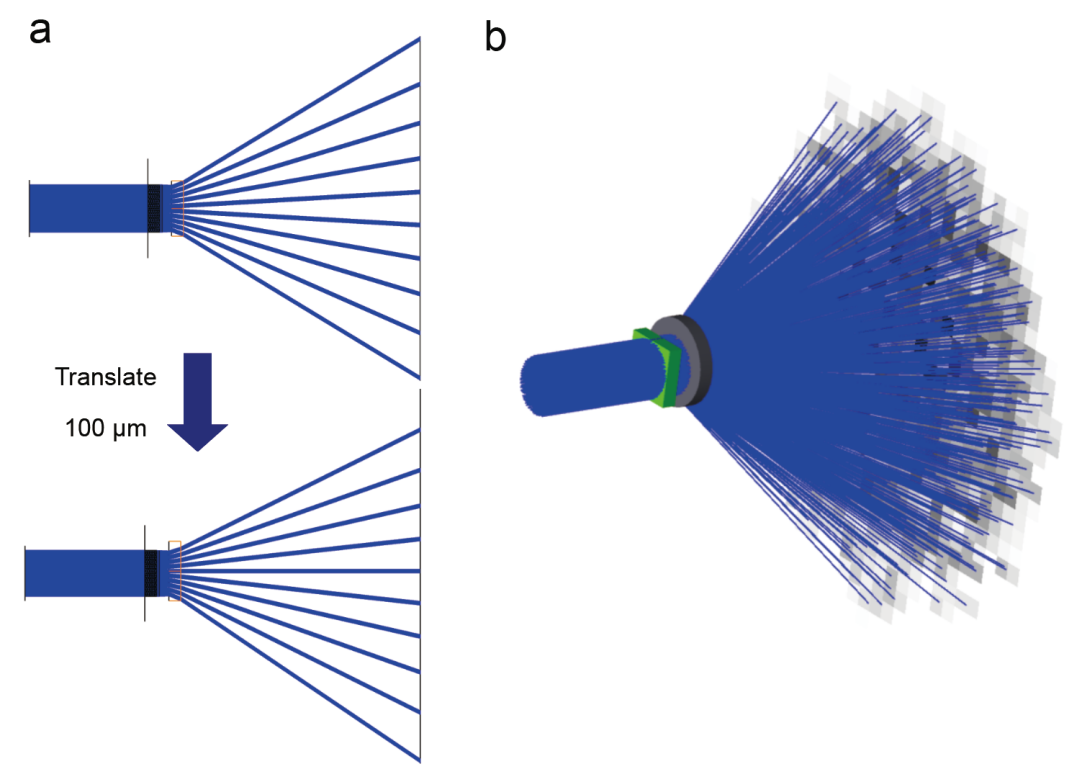


**Fig. S7 | Simulation of dual-mode operation in non-sequential mode. a Beam array scanning mode. b Flash illumination mode.**

Detailed optical system modeling and simulation are required based on Zemax OpticStudio to study the beam projection and scanning mechanisms, providing guidance for experimental testing. By using components including binary surfaces, Boolean operations, and arrays, cascaded metasurfaces are constructed. Geometrical rays are captured by detectors as shown in Fig. S7. In the beam scanning scanning mode, the system achieves a ±35° field of view (FoV) with a 100 μm translational displacement, which aligns with the design specifications. The flash illumination mode demonstrates a broad beam illumination effect, consistent with experimental observations. Although the sequential mode cannot directly construct an array of metasurfaces, it is possible to achieve similar results by invoking non-sequential components.

***2. Simulation of beam array scanning mode***


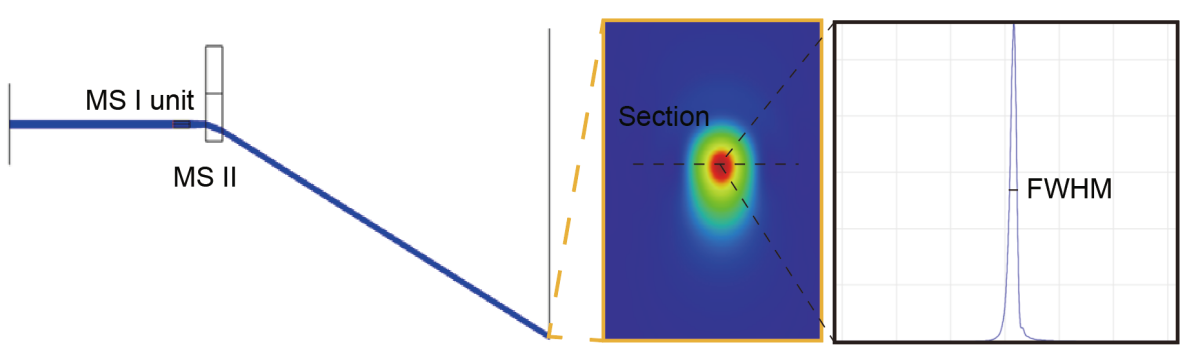


**Fig. S8 | Simulation of the beam array scanning mode in sequential mode.**

In experiments, deformation of the beam spot morphology at the edges is observed, but simulations in non-sequential mode revealed that, based on geometrical ray-tracing alone, the beam spot shape and FWHM showed no significant change. Thus, physical optics simulation is necessary, taking into account that the incident laser beam is a Gaussian beam and considering more complex diffraction effects. Therefore, a simulation model is established in sequential mode as shown in Fig. S8. After conducting physical optics simulations, the FWHM of the beams are measured from the cross-sectional curve. The simulated beam spot morphology closely matches the experimental result, confirming that the simulation approach is fundamentally correct.

**Supplementary** **Note 8: Angle resolution analysis of the beam array scanning mode**


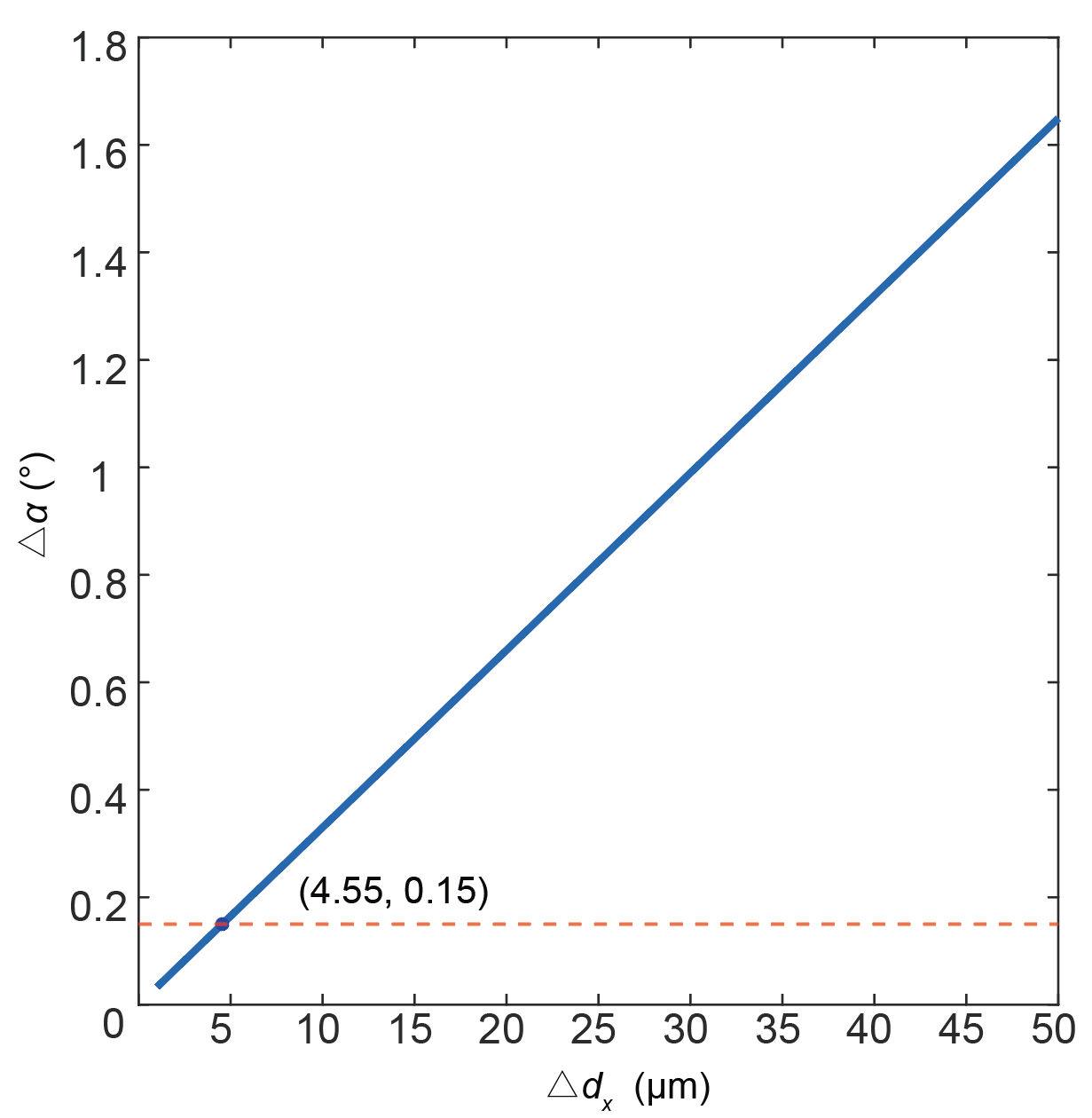


**Fig. S9 | Graph of the relationship between adjustment step size of output deflection angle and displacement step size.**

Equation (6) represents the relationship between the translational step size ∆*d_x_* of MS II and the deflection angle step size ∆*α_j_* of the output beam. Since $\left( \frac{p\lambda_{0}}{\pi}\left( d_{x}-\Gamma_{j} \right) \right)^{2}\ll1$, equation (6) can be approximated as the following equation:

$$\begin{aligned} \Delta\alpha\approx\frac{p\lambda_{0}}{\pi}{\Delta d}_{x}\#\left( s-12 \right) \end{aligned}$$

Based on the above equation, the relationship between the deflection angle step size ∆*α* of the output beam and the translational step size ∆*d_x_* when ∆*d_x_* ranges from 0 to 50 μm can be plotted (as shown by the blue solid line in Fig. S9).

Given that the measured beam divergence angles *δ* in the beam array scanning mode are within 0.15°, it is represented by the red dashed line in Fig. S9, which intersects with the line representing the relationship between the translational step size and the deflection angle step size at the point (4.55, 0.15). Equation (9) expresses the angular resolution in this mode as:

$$\begin{aligned} Re=\max\left( \delta,\Delta\alpha\right)\#\left( s-13 \right) \end{aligned}$$

In summary, it can be deduced that for translational step sizes less than 4.55 μm, *δ* dictates the system’s resolution, while for step sizes greater than 4.55 μm, ∆*α* governs the resolution.

**Supplementary** **Note 9: Theoretical and experimental results of beam coverage in the two-dimensional angular domain**


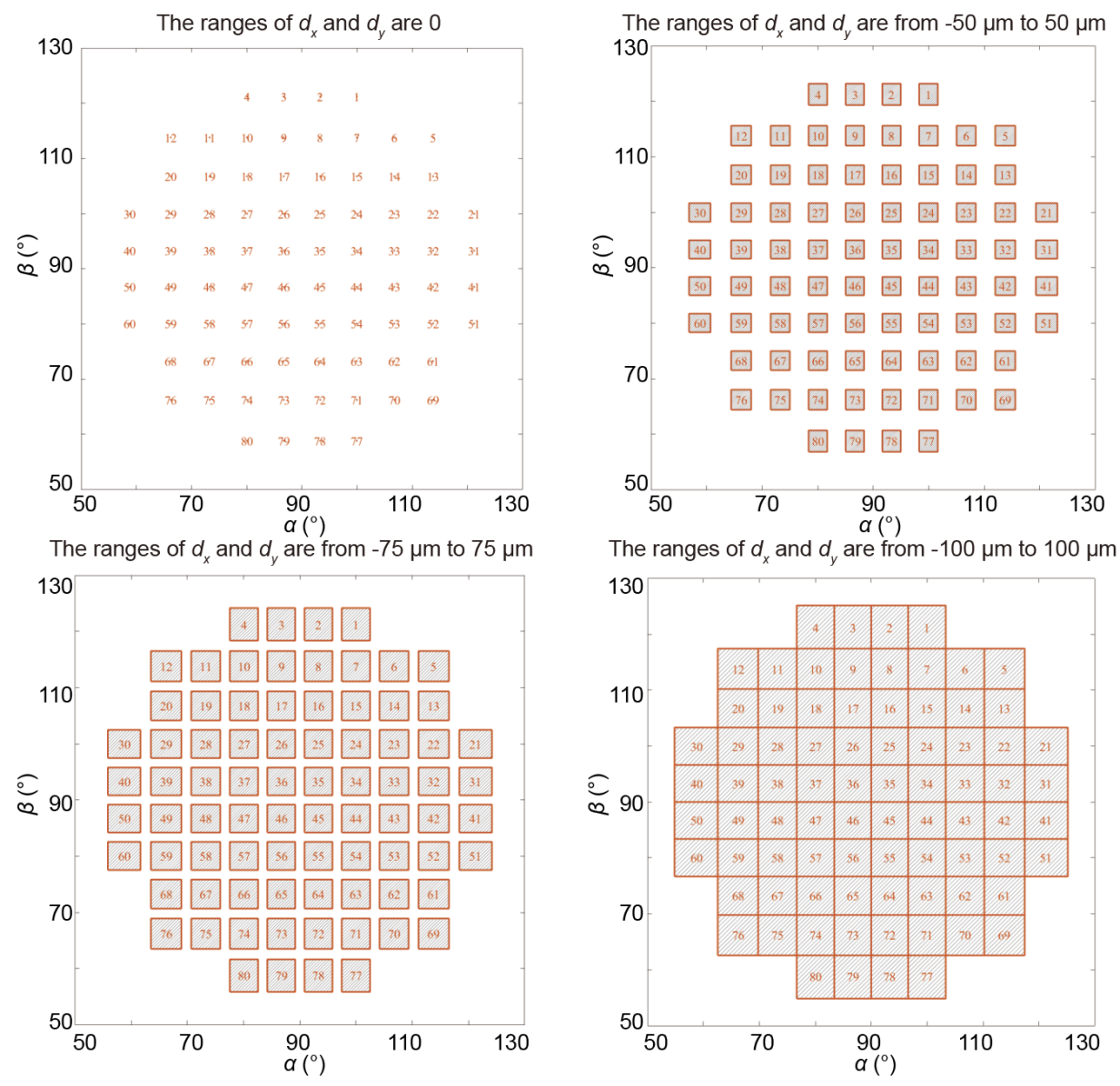


**Fig. S10 | In beam array scanning mode, the schematic diagram of the theoretical beam coverage in the two-dimensional angular domain as a function of translational displacement range.**

This section analyzes the beam coverage capability of device in beam array scanning mode. By incorporating the designed system parameters into Equations (4) and (5), The diagram showing the range covered by the deflection angles of the beam array under different displacement ranges is shown above.

It is evident that when the displacement ranges of *d_x_* and *d_y_* are 0, the directions of the 80 output beams are uniformly distributed within the range of *α* and *β* from 58°-121°. As the displacement ranges *d_x_* and *d_y_* gradually increase, the adjustable ranges of the direction angles of the 80 output beams gradually expand. When the ranges of displacement *d_x_* and *d_y_* are both ±100 μm, the direction angles of the 80 output beams can cover the angular domain range of *α* and *β* from 55°-125° without blind spots.


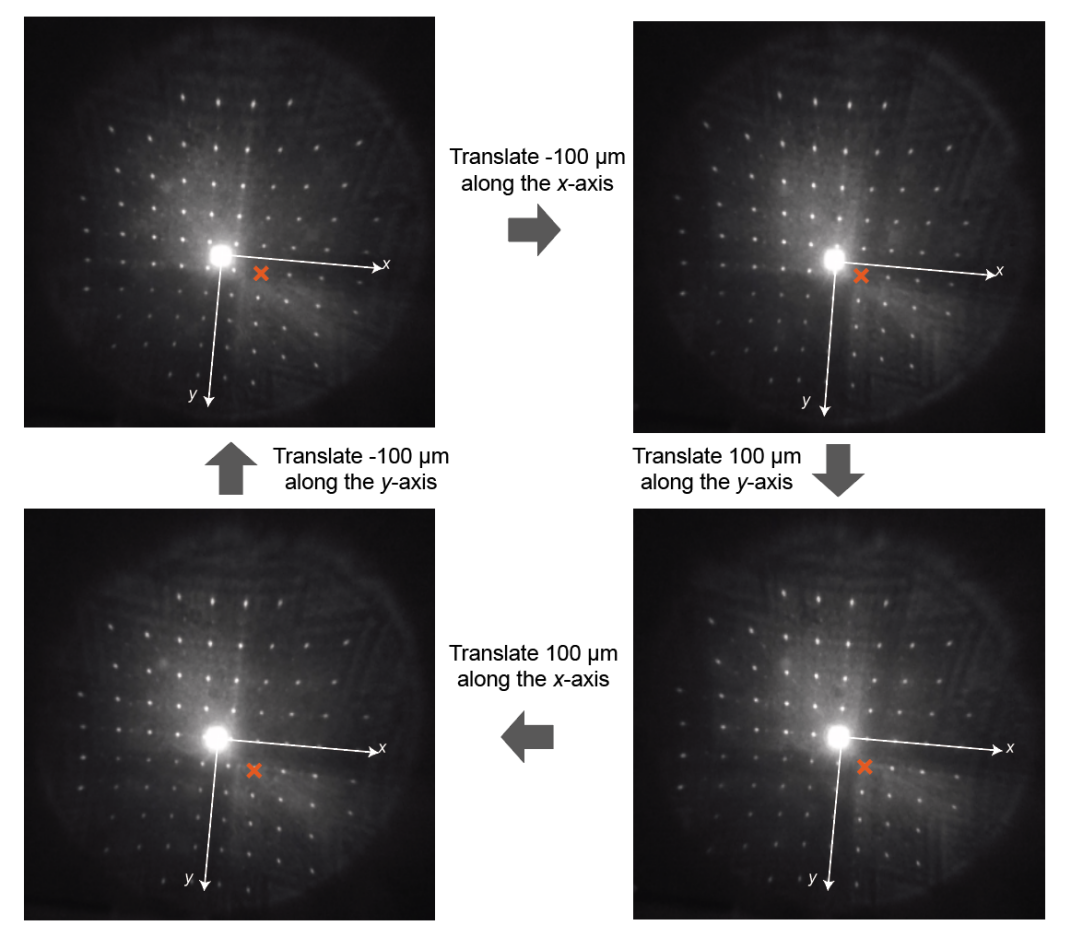


**Fig. S11 | In beam array scanning mode, observing beam scanning experiment on the light screen.**

To verify the beam coverage capability of the device, the beam array is projected onto a light screen for observation. Simultaneously, the SMA micro-actuator is used for scanning in the *x* and *y* directions, as shown in Fig. S11. By measuring the distance from the device to the light screen and the distribution position of the beam spots, the deflection angles of the beam spots can be obtained, which closely matches the theoretical calculations. This indicates that the device designed in this work can achieve full coverage of a ±35° FoV in beam array scanning mode with only a minimal translational displacement of ±100 μm.

**Supplementary** **Note 10: Simulation verification of the uniformization effect of output beams achieved by THCMs in flash illumination mode**


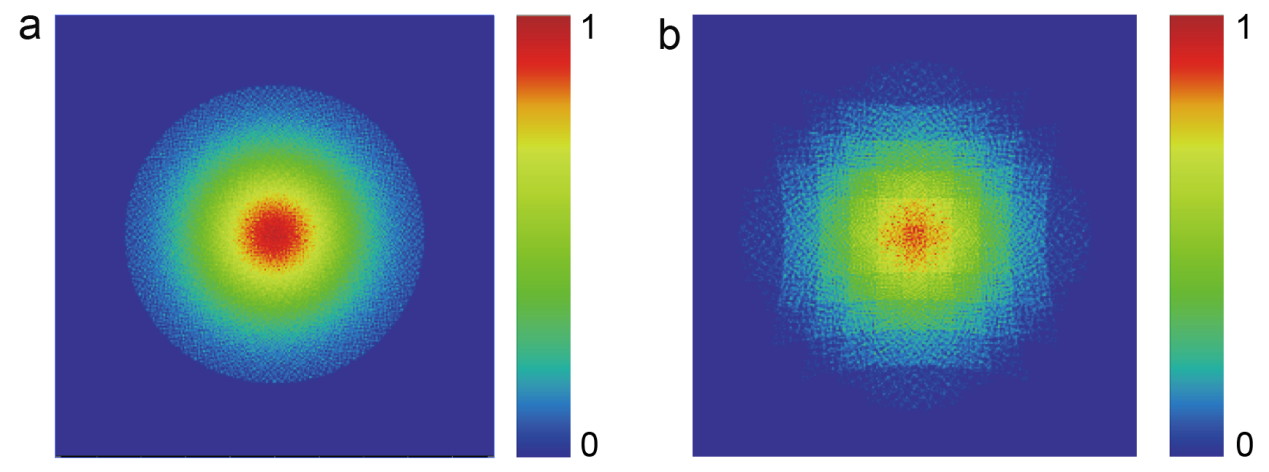


**Fig. S12 | a** Simulation light intensity plot of the output beam when RCP light is incident and only passes through MS II. **b** Simulation light intensity plot of the output beam when RCP light is incident and passes through THCMs.

To simulate and verify the beam uniformity effect in the flash illumination mode, we used a Gaussian light source in Zemax and simulated the light intensity distribution maps 10 cm after the beam output in two scenarios: with only MS II and with THCMs. Comparing the two sub-figures in Fig. S12, it is evident that THCMs can improve the uniformity of the output beam. Furthermore, we calculated the standard deviations of the normalized light intensity in both sub-figures, yielding a standard deviation of 0.2018 for the case with only MS II and 0.1754 for the case with THCMs.

**Supplementary** **Note 11: Testing of transmission efficiency for the beam forming device**

The transmission efficiency of the beam forming device presented in this paper is measured using a GCI-08 optical power meter (Daheng Optics). In flash illumination mode, with an incident light power of 18 mW, the total output power is 7.9 mW, corresponding to an energy utilization efficiency of 43.9%.

In beam array mode, with the same incident light power of 18 mW, the measured beam optical power at a distance of 10 cm emitted from the center *x*-coordinates of different MS I array units is shown in the table below. Excluding the zeroth-order light, based on the rotational symmetry of the beam array mode, a weighted average is calculated from the measured power values at five positions. Finally, the average output power per single beam is calculated to be 42.9 μW, resulting in a total energy utilization efficiency of 20%. The relatively low throughput in our current beam array scanning mode can significantly limit the achievable detection range, especially when optical power is distributed among multiple beamlets.

To address this, we are actively working on two parallel strategies. First, we are improving the optical efficiency of the cascaded metasurfaces. In our current implementation, the relatively low throughput is primarily due to non-ideal fabrication processes, which result in a significant portion of light remaining in the zero-order, thereby reducing the energy distributed to the intended beam array. We are addressing this by iteratively optimizing fabrication parameters, including improving the resolution of electron beam lithography (EBL) and enhancing the lift-off process to reduce residual artifacts. Notably, we have recently fabricated metasurface samples—based on a similar cascaded architecture—that achieved energy throughput exceeding 60%, demonstrating the effectiveness of these improvements. In addition, recent studies have shown that reflective losses and stray diffraction can be further suppressed by increasing transmission through techniques such as anti-reflection coating and by designing suitable substrates^5^. Second, we are exploring the use of VCSEL array sources, where each VCSEL unit is directly aligned with a single unit cell in MS I. This approach would exclude the need to spread the optical power across multiple beamlets from a single source, thereby preserving per-beamlet intensity and enhancing overall system throughput.

**Supplementary Table 1 | Beam optical power at 10 cm emission distance for different *x* coordinates of MS I in beam array scanning mode**

| Emission Position/mm | 0 | 0.1 | 0.3 | 0.5 | 0.7 | 0.9 |
| --- | --- | --- | --- | --- | --- | --- |
| Optical Power/mW | 1.6 | 0.079 | 0.049 | 0.045 | 0.042 | 0.028 |

**Supplementary** **Note 12: Overview of the dual-mode depth detection algorithm based on binocular vision**

***1. Summary of the dual-mode depth detection process***

To facilitate demonstration and simplify the proposed dual-mode LiDAR system, a detection principle based on binocular vision is adopted to verify the capability of this device for 3D detection and its ability to meet the beam projection requirements of LiDAR. Since the detection is based on the visual principle, it is first necessary to calibrate the camera to obtain its intrinsic parameters and perform distortion correction on the captured images.

In flash illumination mode, well-established and widely used stereo vision algorithms can be directly employed for the detection task. The detection process involves several steps: first, image acquisition is performed using the left and right cameras; second, stereo matching algorithms are applied to generate a disparity map; third, depth information is calculated based on the disparity map; finally, the translational step size in the beam array scanning mode is determined according to the region of interest.


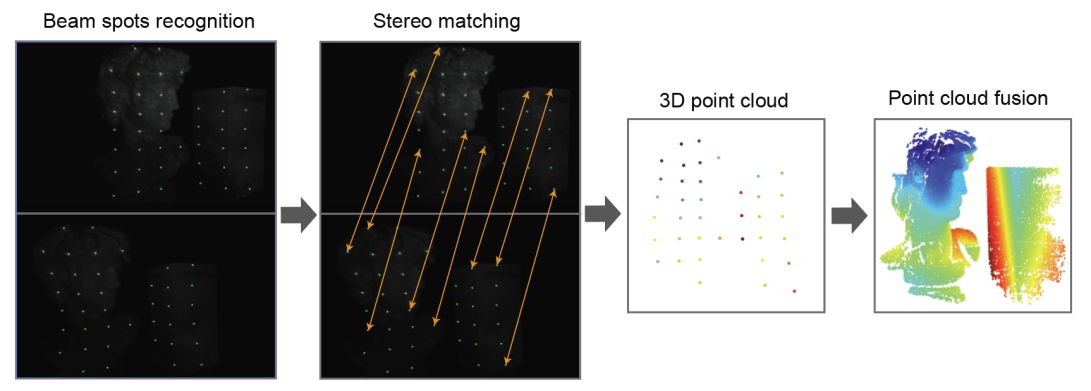


**Fig. S13 | Flowchart of detection process based on binocular vision in beam array scanning mode**.

For the beam array scanning mode, the detection process is illustrated in Fig. S13. First, a sliding window algorithm is used to identify beam spots in the image, yielding the positional coordinates of the beam spot array. The sliding window algorithm scans the image using a window of a fixed size. A simple global threshold or basic adaptive threshold may mistakenly identify stray light around the beam spots as objects. Therefore, employing the sliding window algorithm helps to detect beam spots with greater accuracy and lower computational cost, while minimizing the interference from stray light.

Next, the Coherent Point Drift (CPD) algorithm is employed, which treats the stereo matching problem of the beam spots as the alignment of probability density functions. The optimal pairing is achieved by minimizing the difference in probability distribution between two spot sets from the left and right perspectives. The CPD algorithm offers two types of matching: rigid and non-rigid. Rigid matching only allows for translation, rotation, and scaling of the beam spot array, thus preserving its topological structure. However, since the internal topology of the beam spot array can deform under different perspectives, rigid matching alone cannot achieve full alignment. In non-rigid matching, each spot’s coordinates can transform freely, enabling full alignment between the two beam spot arrays, but it often compromises the topology of the point cloud, resulting in mismatches between spots. Therefore, a two-step approach is chosen: rigid matching is first applied to achieve coarse alignment of the beam spot arrays, followed by non-rigid matching for fine alignment. This method preserves the topological structure of the beam spot arrays while achieving effective left-right array matching. Once the beam spot arrays from the left and right cameras are matched, the 3D point cloud can be obtained using the principle of triangulation. Finally, the targeted object is scanned by the beam array at a set step size, resulting in an adjustable-resolution 3D point cloud.

***2. Principle of depth detection based on binocular vision***


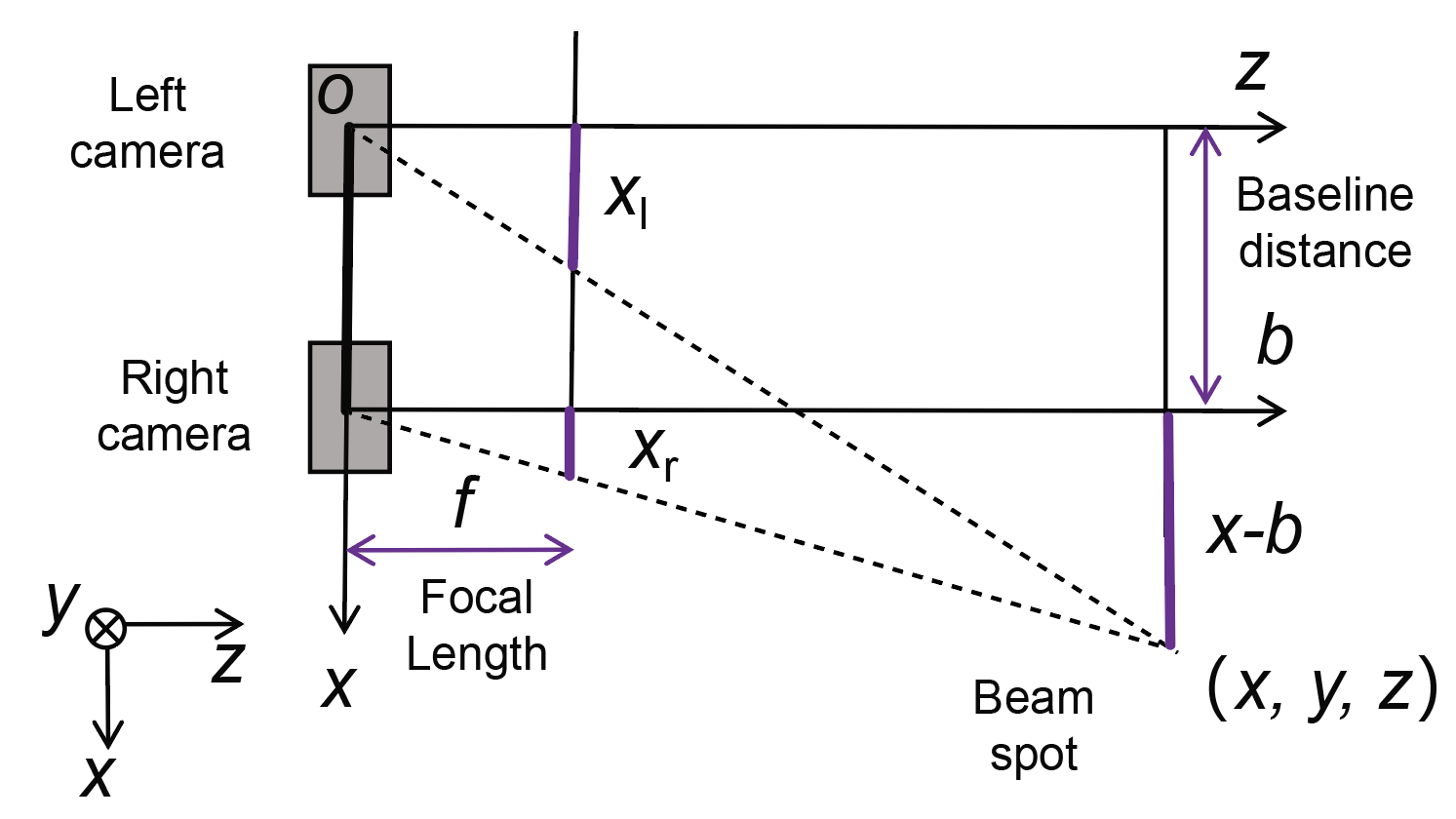


**Fig. S14 | The schematic diagram of the** **binocular vision detection principle.**

In binocular vision detection, cameras are placed at different positions, mimicking the two human eyes, to capture two images with parallax. Parallax refers to the relative positional difference of beam spots in the two images due to different viewing angles. By computing and analyzing this parallax, the spatial position of each beam spot, i.e., its depth information, can be inferred. In this experimental system, the centers of the left and right cameras are adjusted to the same horizontal line, and the line connecting the centers of the two cameras is parallel to the dual-mode beam forming device, facilitating the simplification of subsequent depth calculations.

In the spatial Cartesian coordinate, the center of the left camera is set as the origin of the reference coordinate system, with the *x*-axis representing the horizontal direction and the *y*-axis representing the vertical direction. The *x*-*y* plane is parallel to the surface of the superstructure, and the distance between the left and right cameras is the baseline distance, denoted as *b*. Assuming a beam reflects off the surface of an object and has coordinates (*x*_l_, *y*_l_) on the focal plane of the left camera and (*x*_r_, *y*_r_) on the focal plane of the right camera, the formula for calculating the spatial coordinates (*x*, *y*, *z*) of this beam spot can be derived based on the similarity of triangles in the diagram as follows:

$$\begin{aligned} z=\frac{b\times f}{x_{l}-x_{r}}\#\left( s-14 \right) \end{aligned}$$

$$\begin{aligned} x=\frac{z\times x_{l}}{f}\#\left( s-15 \right) \end{aligned}$$

$$\begin{aligned} y=\frac{z\times y_{l}}{f}\#\left( s-16 \right) \end{aligned}$$

In practice, image coordinates are typically given in pixel coordinates. Let (*x’*_l_, *y’*_l_) denote the pixel coordinates of the beam spot obtained by the left camera, and (*x’*_r_, *y’*_r_) denote the pixel coordinates of the same spot obtained by the right camera. To recover the actual position of the object in space from these pixel coordinates, it is crucial to convert these pixel coordinates (*x’*_l_, *y’*_l_) and (*x’*_r_, *y’*_r_) to the actual imaging coordinates (*x*_l_, *y*_l_) and (*x*_r_, *y*_r_) of the cameras. This transformation process requires determining the intrinsic parameters of the cameras, which describe the imaging characteristics, including focal length, principal point position, and distortion coefficients.

***3. Camera calibration***


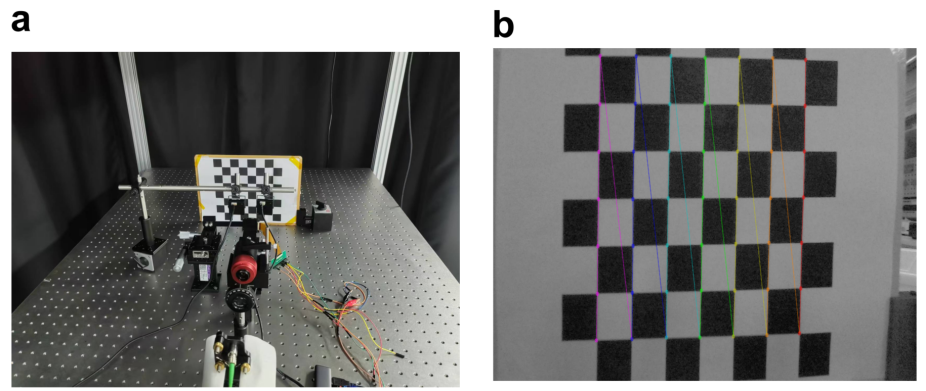


**Fig. S15 | Using a chessboard for** **camera calibration. a** Experimental system. **b** Chessboard recognition image.

Before performing depth detection, it is necessary to complete a preliminary task: obtaining the camera’s intrinsic parameters through a chessboard calibration method. During this calibration process, multiple images of a chessboard placed at various positions are captured using both the left and right cameras. The chessboard is positioned to cover the entire FoV as comprehensively as possible, ensuring accurate estimation of the intrinsic parameters.

**Supplementary** **Note 13: Accuracy evaluation of 3D detection results in beam array scanning mode**


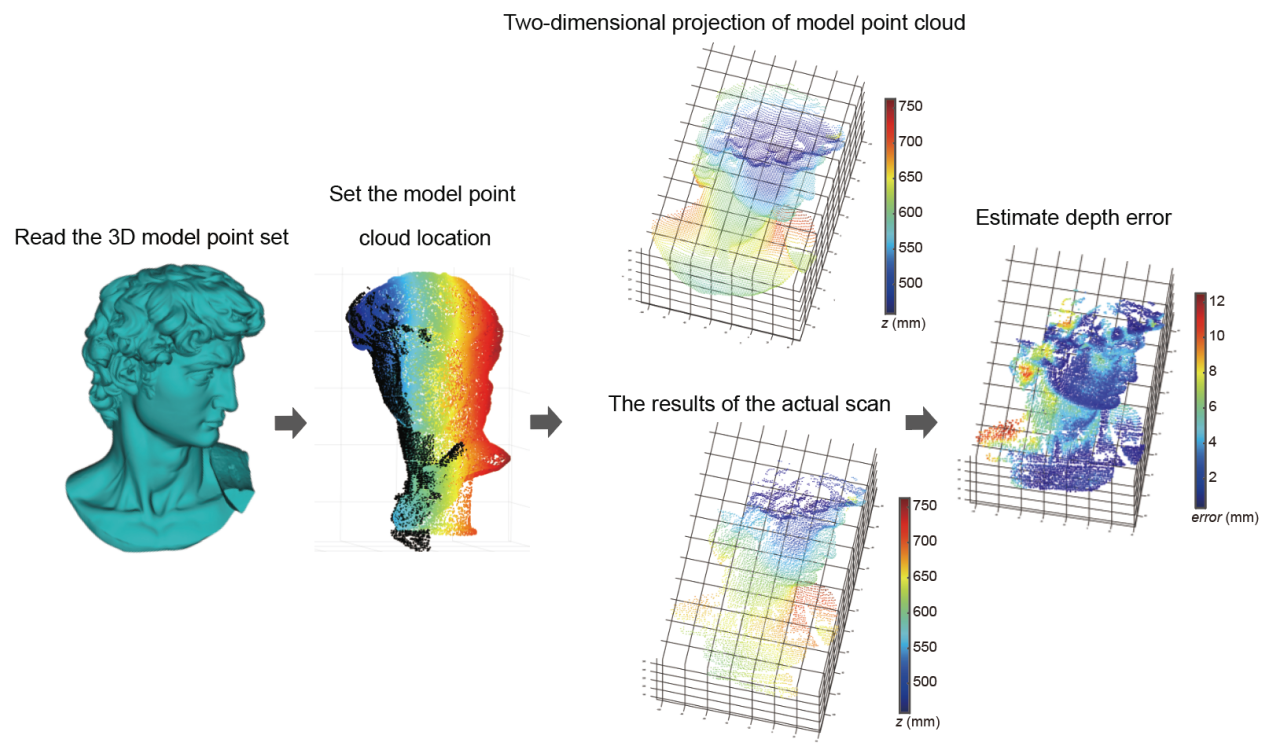


**Fig. S16 | Flowchart of accuracy evaluation process for 3D detection point clouds.**

To evaluate the accuracy of the 3D point cloud obtained in beam array scanning mode with a translational step size of 10 μm on the MS II, the existing 3D model of the David plaster statue is used as the ground truth for comparison. This 3D model was constructed based on precise measurement data taken during the original production, ensuring its reliability as a true value reference.

The evaluation process involved several key steps. Firstly, the 3D model is imported, and the model’s point cloud is extracted. Then, the point cloud’s pose is adjusted according to the experimental setup. Next, voxel projection and k-nearest neighbor (k-NN) filtering are applied to denoise and project the model’s point cloud. After that, the point cloud obtained from scanning is compared with the model’s point cloud by finding the closest (*x*, *y*) coordinates and analyzing the differences in the *z*-axis depth to calculate accuracy. The results show an average deviation of 4.0729 mm, with a standard deviation of 2.5376 mm, a maximum deviation of 12.6928 mm, and a minimum deviation of 0.0837 mm. Based on the model point cloud’s maximum *z*-value of 760 mm, the overall error is below 1.02%. These results demonstrate the high accuracy of the 3D point cloud, and based on the earlier analysis of angular resolution, it is evident that reducing the translational step size further could yield even higher-density 3D point cloud images.

**Supplementary** **Note 14:** **Comparison of beam array scanning mode with advanced metasurface-based 3D detection methods**

In the proposed beam array scanning mode of the THCMs, when the cascaded metasurfaces remain stationary, it can achieve beam array projection with small divergence angles across a wide FoV. High-resolution 3D detection with precise 2D angular domain coverage can be achieved through small displacements between the metasurfaces. Table 2 lists key features of the beam array scanning mode and compares them with existing advanced metasurface-based 3D detection approaches, emphasizing the balanced advantages of our proposed method.

The lightweight structure and high-precision beam manipulation capability of metasurfaces make them promising core components for realizing miniaturized LiDAR systems. Existing approaches based on metasurfaces for dynamic beam scanning can be categorized into two types. The first is all-solid-state beam scanning that does not require moving components but is limited by both the FoV and beam divergence. The second method utilizes small motions of the metasurfaces to achieve semi-solid-state beam scanning. By taking full advantage of the metasurface's planar properties, our approach enables high-quality multi-beam projection over a wide FoV, while maintaining significant design flexibility.

Existing research on static mutil-beam projection via metasurfaces demonstrates efficient wide FoV 3D detection, where the resolution is mainly determined by the sampling angle. However, these methods suffer from the inherent trade-off between resolution and detection range. In contrast, the resolution of our beam array scanning mode is governed by beam divergence, and our design allows for the potential to further expand the FoV and increase the number of beam projections, thereby minimizing translational displacement and improving detection efficiency.

**Supplementary Table 2 | Comparison of beam array scanning mode and metasurface-based 3D detection approaches**

|  | Our work | [^6^] | [^7^] | [^8^] | [^9^] | [^10^] | [^11^] | [^12^] | [^13^] |
| --- | --- | --- | --- | --- | --- | --- | --- | --- | --- |
| Detection approach | Semi-solid-state beam scanning | Semi-solid-state beam scanning | All-solid-state beam scanning | All-solid-state beam scanning | Static multi-beam projection | Static multi-beam projection | Static multi-beam projection | Static multi-beam projection | Static multi-beam projection |
| Beam number | 80 | 25 | 1 | 1 | 45.7k | 9 | 10k | 69 | 6k |
| Wavelength/nm | 1064 | 1064 | 1560 | 655 | 940 | 980 | 633 | 940 | 633 |
| FoV/° | 70 | 30 | 15 (1D) | 22 (1D) | 158 | 20 | 180 | 120 | 76 |
| Resolution/° | 0.15 | 0.22 | 0.6 | - | 0.611 | 10 | 1.757 | 15 | 0.22 |
| Material | a-Si | a-Si | Au | TiO2 | GaAs | GaAs | a-Si | a-Si | a-Si |

**Supplementary** **Note 15: A dual-mode beam forming device**


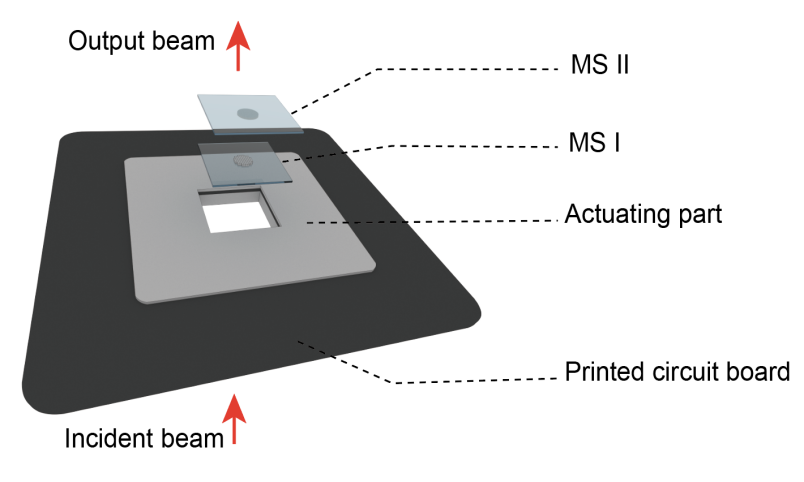


**Fig. S17 | Exploded schematic of the dual-mode beam forming device.**

We integrate the fabricated THCMs with the SMA micro-actuator to achieve a dual-mode beam forming device, which is shown in Fig. S18. MS I remains fixed, while the actuator drives MS II to perform slight translational movements in the *x*-*y* plane, enabling beam array mode beam scanning. The actuator is a customized version of a commercial sensor shift optical image stabilization device (GCDS01A, GalaxyCore). It can accommodate two metasurfaces with a spacing of 500 μm, allowing the incident beam to pass through. At the output, a metallic circular aperture blocks incident light that has not passed through the metasurface. The actuator is made from shape memory alloy (SMA) material in the form of a wire. The expansion and contraction characteristics of the SMA material, based on electrothermal principles, generate motion. This motion is controlled by a driving circuit and software algorithm that adjust the wire length to achieve translation in the *x*-*y* plane.

In this work, the designed metasurface translational range is ±100 μm with a step size of 10 μm, while the actuator has a translational range of ±150 μm and a minimum step size of 3 μm, fully meeting the design requirements. Including the printed circuit board (PCB), the actuator has dimensions of 4 cm × 4 cm × 0.5 cm and weighs 26 g. Future research aims to integrate new light sources, such as vertical-cavity surface-emitting lasers (VCSEL), to further achieve a high-performance, highly integrated, light weight, and environment-adaptive miniaturized LiDAR system.

The scanning frequency or frame rate is highly dependent on the requirements of practical applications. For common scenarios like dynamic obstacle avoidance, we believe that a ~10 Hz frame rate is achievable. Assuming a square-shaped obstacle with a side length of *L*_ob_ is placed in front of the LiDAR system with a distance of *Dis*_ob_, and four points are needed to detect the obstacle (Fig. S18a). Therefore, the necessary angular resolution can be calculated by:

$$\begin{aligned} Re=\frac{L_{\mathrm{ob}}}{{Dis}_{\mathrm{ob}}} \end{aligned}$$

Based on equation (6) in the main text, the required scanning step size of the actuator for each detection frame is:

$$\begin{aligned} {\Delta d}_{y}=\frac{\pi}{\left| p \right|\lambda_{0}}Re=\frac{\pi L_{\mathrm{ob}}}{\left| p \right|\lambda_{0}{Dis}_{\mathrm{ob}}} \end{aligned}$$

If the SMA actuator drives the metasurface MS II along a simple S-shape track (**Figure 1a** in the main text), the total number of actuation cycles along *x*-axis for each detection frame is:

$$\begin{aligned} Num=\left[ \frac{P_{m}}{{\Delta d}_{y}} \right]=\left[ \frac{{\left| p \right|\lambda_{0}{Dis}_{\mathrm{ob}}P}_{m}}{\pi L_{\mathrm{ob}}} \right] \end{aligned}$$

where [ ] represents the round down function to obtain an appropriate integer number.

We assume *Dis*_ob_ = 50 m. Given the parameter values *p* = -1700 mm^-2^, *λ*_0_ = 1064 nm, *P*_m_ = 200 μm, and the experimentally characterized duration of a single actuation cycle of 200 μm as 18.2 ms, the beam scanning frequency can be obtained (Fig. S18b). This indicates that the detection frame rate can achieve >10 Hz (<100 ms) for a 1-m obstacle positioned in a 50 m distance. If the same angular resolution of ~0.3° is required as the main text, which corresponds to a *L*_ob_ = 0.3 m, a total frame rate of ~3 Hz is achievable.


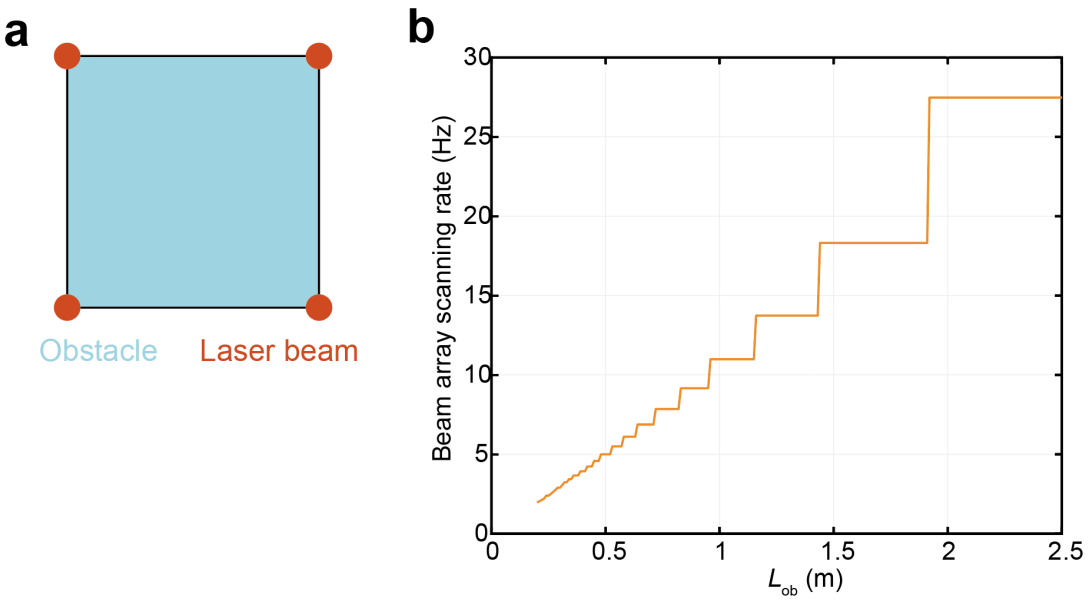


**Fig. S18** | The obstacle avoidance performance of the beam array scanning mode of the proposed LiDAR system.

Moreover, it is worth noting that the actuator is responsible for providing a dynamic beam array and coded structural light, and the 3D reconstruction is based on the binocular disparity in our system. Therefore, the specific direction angles of the beam array are not incorporated in the reconstruction procedure, and the actuation precision of the actuator doesn’t affect the precision of the obtained depth map. This indicates that the actuator doesn’t need a complex feedback system, which is an advantage of our proposed device.

The SMA actuation mechanism is renowned for its reliability and large actuation range, making it a popular choice in smartphone optical image stabilization systems. However, for scenarios requiring higher frame rates, alternative mechanisms such as electromagnetic or piezoelectric actuation may be more suitable. Additionally, optimizing the scanning trajectory can further reduce the frame time, thereby enhancing system performance.

**Supplementary** **Note 16: Metasurface fabrication process**


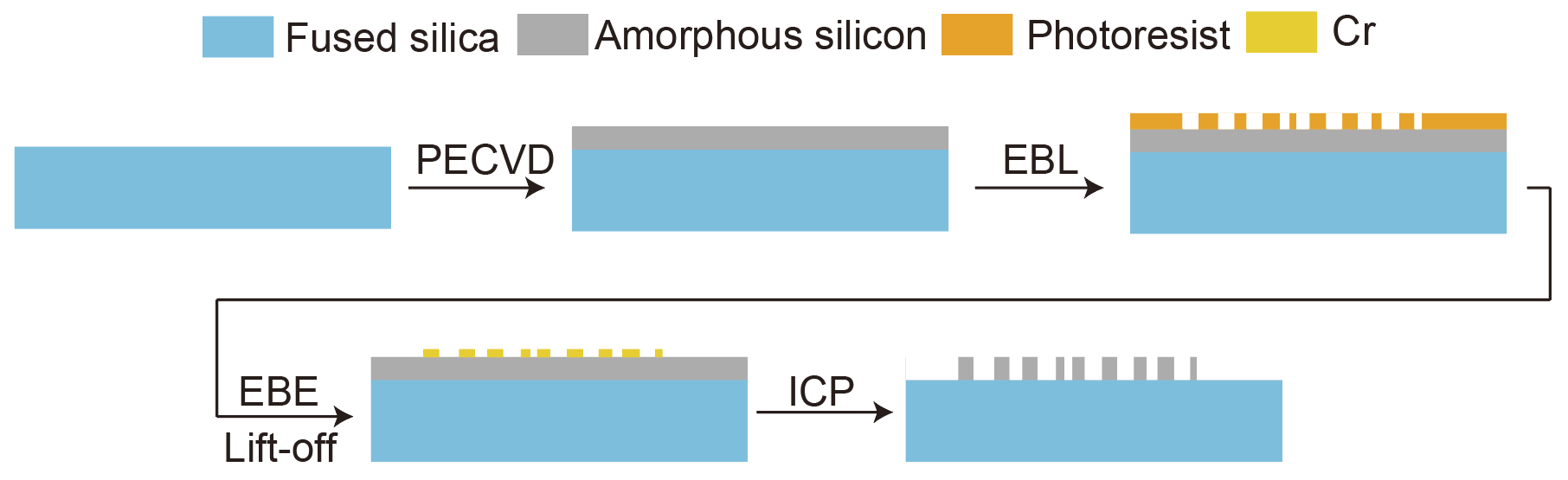


**Fig. S19 | Metasurface fabrication process diagram.**

The metasurface fabrication process consists of several key steps utilizing standard semiconductor micro/nanofabrication techniques. Initially, a 500 μm thick, 4-inch fused silica glass wafer is laser-cut into small square pieces (10×10 mm^2^). Subsequently, a 600 nm amorphous silicon film is deposited via plasma-enhanced chemical vapor deposition (PECVD), and its thickness along with dispersion properties is characterized using ellipsometry. Following this, a layer of positive photoresist (ZEP520A) is spin-coated, and electron-beam lithography (EBL) is employed to transfer the designed pattern onto the photoresist. Next, a 50 nm chromium film is deposited using electron-beam evaporation (EBE), and a lift-off process is conducted to form a patterned chromium mask, which serves as a hard mask for the subsequent etching. Finally, inductively coupled plasma (ICP) etching is applied to etch through the amorphous silicon film, and the chromium mask is removed, resulting in the completed metasurface.

Observation under a scanning electron microscope (SEM) shows that the surface morphology remains intact (Fig. 3a), with the linewidth error controlled within ±10 nm. Furthermore, the process experiment sample, cut synchronously using a focused ion beam (FIB) and observed under SEM, reveals that the columnar structure is well-preserved, with a height error within ±5 nm. These observations demonstrate the process capability of the adopted metasurface fabrication method. According to the calculated results, the transmission phase shift caused by the ±10 nm in-plane linewidth error does not exceed 30°.

**Supplementary Note 17: Analysis of far-field coupled cascaded metasurfaces based on cascaded scattering matrices**

An analysis of the cascaded scattering matrices can provide a rigorous and comprehensive description of the cascaded metasurface scheme (Fig. S20). For a cascaded metasurface unit cell composed of unit cells of MS I and MS II with an air layer in-between, it is always possible to establish a rectangular coordinate system such that the *x*-axis aligns with the direction of *L_x_*. Under this configuration, the cross-polarization responses (*x*-polarization input and *y*-polarization output, or vice versa) are zero^14^. This indicates that the two polarization channels operate independently and can be examined separately. Without loss of generality, we focus solely on one channel for our analysis.

Since the unit cells of MS I and MS II are linear, time-invariant and reciprocal, their scattering matrices are established as:

$$\left[ \begin{matrix} E_{I}^{1} \\ E_{I}^{2} \end{matrix} \right]=\left[ \begin{matrix} r_{I1} & t_{I} \\ t_{I} & r_{I2} \end{matrix} \right]\left[ \begin{matrix} E_{I}^{10} \\ E_{I}^{20} \end{matrix} \right], \boldsymbol{S}_{\mathbf{MSI}}=\left[ \begin{matrix} r_{I1} & t_{I} \\ t_{I} & r_{I2} \end{matrix} \right]$$

$$\left[ \begin{matrix} E_{\mathrm{II}}^{1} \\ E_{\mathrm{II}}^{2} \end{matrix} \right]=\left[ \begin{matrix} r_{\mathrm{II}1} & t_{\mathrm{II}} \\ t_{\mathrm{II}} & r_{\mathrm{II}2} \end{matrix} \right]\left[ \begin{matrix} E_{\mathrm{II}}^{10} \\ E_{\mathrm{II}}^{20} \end{matrix} \right], \boldsymbol{S}_{\mathbf{MSII}}=\left[ \begin{matrix} r_{\mathrm{II}1} & t_{\mathrm{II}} \\ t_{\mathrm{II}} & r_{\mathrm{II}2} \end{matrix} \right]$$

where *E* represents the complex amplitude of the input or output wave, *r* represents the reflection coefficient, and *t* represents the transmission coefficient. Since the air layer only provides a transmissive phase delay, it is described as:

$$\left[ \begin{matrix} E_{A}^{1} \\ E_{A}^{2} \end{matrix} \right]=\left[ \begin{matrix} 0 & e^{i\delta_{d}} \\ e^{i\delta_{d}} & 0 \end{matrix} \right]\left[ \begin{matrix} E_{A}^{10} \\ E_{A}^{20} \end{matrix} \right], \boldsymbol{S}_{\mathbf{Air}}=\left[ \begin{matrix} 0 & e^{i\delta_{d}} \\ e^{i\delta_{d}} & 0 \end{matrix} \right]$$

where *δ*_d_ = 2π × Distance/*λ*.

Following the scattering matrix cascading approaches described in the literature^15,16^, the scattering matrix of the cascaded metasurface unit cell is obtained:

$$\boldsymbol{S}_{\mathbf{total}}=\left[ \begin{matrix} r_{total1} & t_{\mathrm{total}} \\ t_{\mathrm{total}} & r_{total2} \end{matrix} \right],\left\{ \begin{matrix} r_{total1}=r_{I1}+\frac{r_{\mathrm{II}1}t_{I}^{2}e^{i2\delta_{d}}}{1-r_{I2}r_{\mathrm{II}1}e^{i2\delta_{d}}} \\ t_{\mathrm{total}}=\frac{t_{I}t_{\mathrm{II}}e^{i\delta_{d}}}{1-r_{I2}r_{\mathrm{II}1}e^{i2\delta_{d}}} \\ r_{total2}=r_{\mathrm{II}2}+\frac{r_{I2}t_{\mathrm{II}}^{2}e^{i2\delta_{d}}}{1-r_{I2}r_{\mathrm{II}1}e^{i2\delta_{d}}} \end{matrix} \right.$$

Given this expression, it can be inferred that $r_{total1}\approx0$, $r_{total2}\approx0$ and $t_{\mathrm{total}}\approx t_{I}t_{\mathrm{II}}e^{i\delta_{d}}$ when reflection of unit cells of MS I and MS II is negligible and the majority of the optical power is transmitted. Since the unit cell geometries are selected to maximize transmission and minimize reflection for constructing our metasurface unit cell library, this condition is inherently satisfied. Additionally, it is important to note that the aforementioned derivation becomes invalid if near-field coupling effects exist between the unit cells of MS I and MS II, particularly when the distance between them is exceedingly small.

To demonstrate these conclusions more clearly, an additional full-wave simulation was conducted on the cascaded unit cell (Fig. S19). The transmission coefficients *t*_total_ were calculated across various diameter *D* and distance values. The results indicate that the amplitude and phase remain stable when the distance exceeds 200 nm. Moreover, the amplitude errors ||*t*_total_|-|*t*_I_×*t*_II_|| and phase errors |arg(*t*_total_)-arg(*t*_I_×*t*_II_)| are presented. For distances greater than 200 nm and *D* values far from the resonance peaks, the amplitude errors remain below 0.015, and the phase errors remain below 1°. Given that *D* values near the resonance peaks were excluded during the construction of the unit cell library due to their low transmission amplitudes, it is reasonable to conclude that near-field coupling effects in cascaded metasurfaces can be neglected when the distance between layers exceeds 200 nm, and transmission coefficient satisfies *t*_total_ ≈ *t*_I_×*t*_II_ (|*t*_total_|≈|*t*_I_×*t*_II_| and arg(*t*_total_)≈arg(*t*_I_)+ arg(*t*_II_)), leading to Equation (3) in the main text.


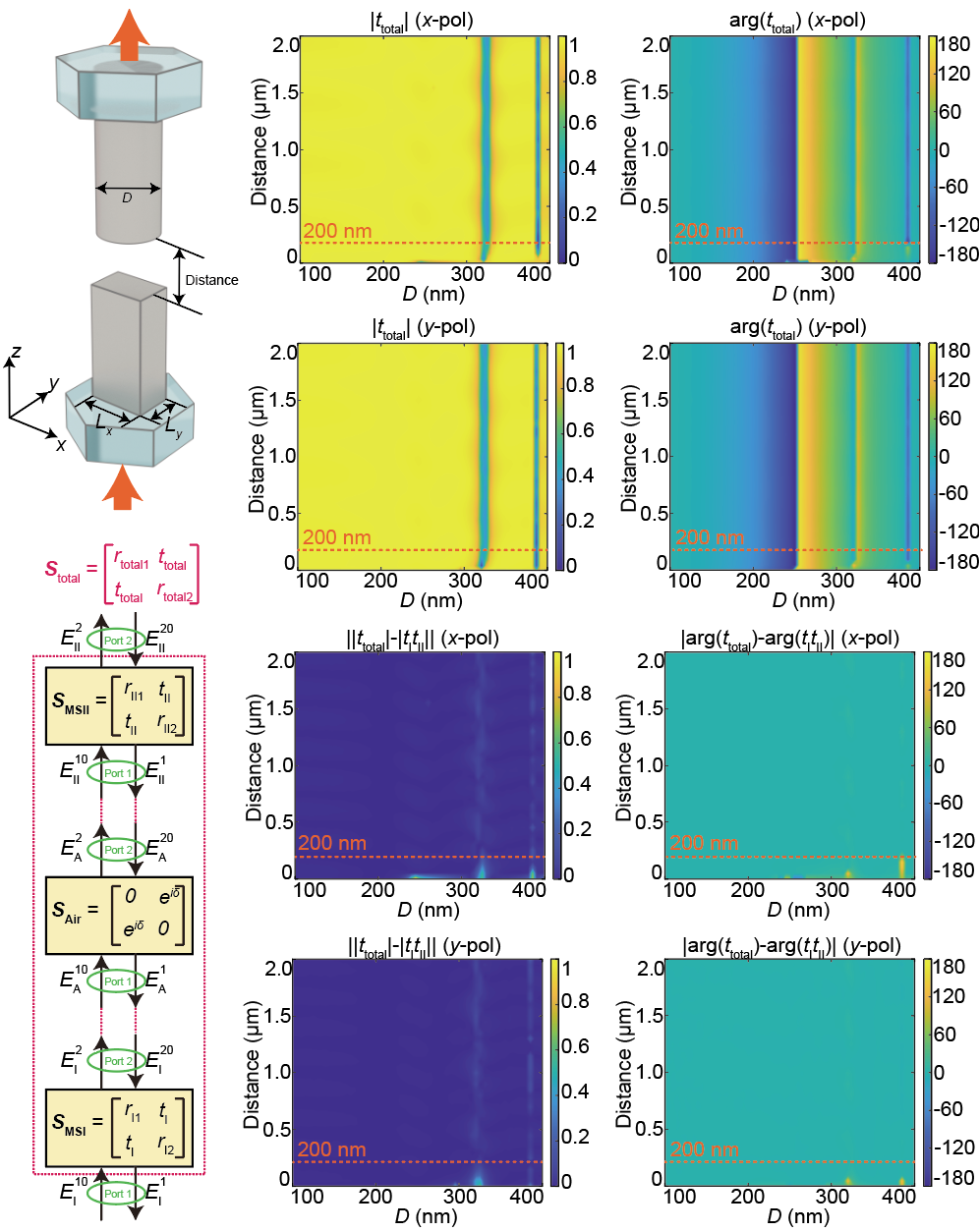


**Fig. S20** | The schematic of the cascaded unit cell and its scattering matrices model. The simulated results of transmission coefficients *t*_total_ of the cascaded unit cell, and calculated amplitude errors ||*t*_total_|-|*t*_I_×*t*_II_|| and phase errors |arg(*t*_total_)-arg(*t*_I_×*t*_II_)| are presented.

However, for distance values exceeding tens of micrometers, the diffraction phenomenon becomes significant, resulting in *t*_total_ ≠ *t*_I_×*t*_II_ due to the wave propagation between layers despite the absence of near-field effects.

**Supplementary Note 18: The maximum field of view of this cascading scheme**

We investigated the maximum field of view (*FoV*_max_) of this cascading scheme as follows. As presented in the main text, the direction angles of the output beam array can be calculated as: $\alpha_{j}\left( d_{x} \right)=\arccos\left( \frac{p\lambda_{0}}{\pi}\left( d_{x}-\Gamma_{j} \right) \right),$ $\beta_{i}\left( d_{y} \right)=\arccos\left( \frac{p\lambda_{0}}{\pi}\left( d_{y}-\Gamma_{i} \right) \right),$ where *α* and *β* represent the direction of the output beam relative to the *x*- and *y*-axes, respectively, *d_x_* and *d_y_* denote the lateral displacements, and *Γ* is the center position of each array unit of metasurface MS I. Besides, parameters *i* and *j* index each array unit. Without loss of generality, we assume *p*<0. Due to the tunable beam steering functionality of the THCMs (tunable hybrid cascaded metasurfaces), the edge of the field of view can be characterized by the outmost beam of the beam array ($\Gamma_{1}=\frac{1-n_{m}}{2}P_{m}$) when the maximum lateral displacement ($d_{x}=\frac{1}{2}P_{m}$) is achieved, which is:

$$\begin{aligned} FoV=2\left( \arccos\left( \frac{p\lambda_{0}}{\pi}\frac{n_{m}P_{m}}{2} \right)-90^{\circ} \right)\#\left( s-17 \right) \end{aligned}$$

Since the radii of the metasurfaces (*r*_1_ for MS I and *r*_2_ for MS II) satisfy $r_{1}=\frac{1}{2}n_{m}P_{m}=\frac{n_{m}}{n_{m}+1}r_{2}$, the *FoV* can be reformulated as:

$$\begin{aligned} FoV=2\left( \arccos\left( \frac{n_{m}}{n_{m}+1}\frac{p\lambda_{0}}{\pi}r_{2} \right)-90^{\circ} \right)\#\left( s-18 \right) \end{aligned}$$

According to Supplementary Note·4, the size of MS II is limited by $r_{2\max}=\frac{\pi}{PM_{\min}\left| p \right|}$. Here *M*_min_ is the minimum number of unit cells used to sample a phase variation of 2*π* at the metasurface edge, and *P* is the metasurface unit cell period. The maximum *FoV* is then calculated by:

$$\begin{aligned} {FoV}_{\max}=2\left( \arccos\left( -\frac{n_{m}}{n_{m}+1}\frac{\lambda_{0}}{PM_{\min}} \right)-90^{\circ} \right)\#\left( s-19 \right) \end{aligned}$$

This indicates that the *FoV*_max_ is mainly determined by the phase sampling capability factors $\frac{\lambda_{0}}{P}$ and *M*_min_. In the proposed design, $\frac{\lambda_{0}}{P}=\frac{1.064}{0.56}=1.90$, *M*_min_ = 3 and *n_m_* = 10, which results in *FoV*_max_ = 70° (±35°). These values are selected as a proof of concept for the fabrication ease. However, state-of-the-art semiconductor fabrication techniques allows larger $\frac{\lambda_{0}}{P}$ and the Nyquist theorem simply requires *M*_min_ ＞ 2, which makes it possible to realize a much larger *FoV*_max_ (Fig. S21). For example, when $\frac{\lambda_{0}}{P}=\frac{1.064}{0.5}=2.13$ and *M*_min_ = 2.1, the *FoV*_max_ = 134° (±67°), which is enough for most LiDAR applications and will be demonstrated in our future work.


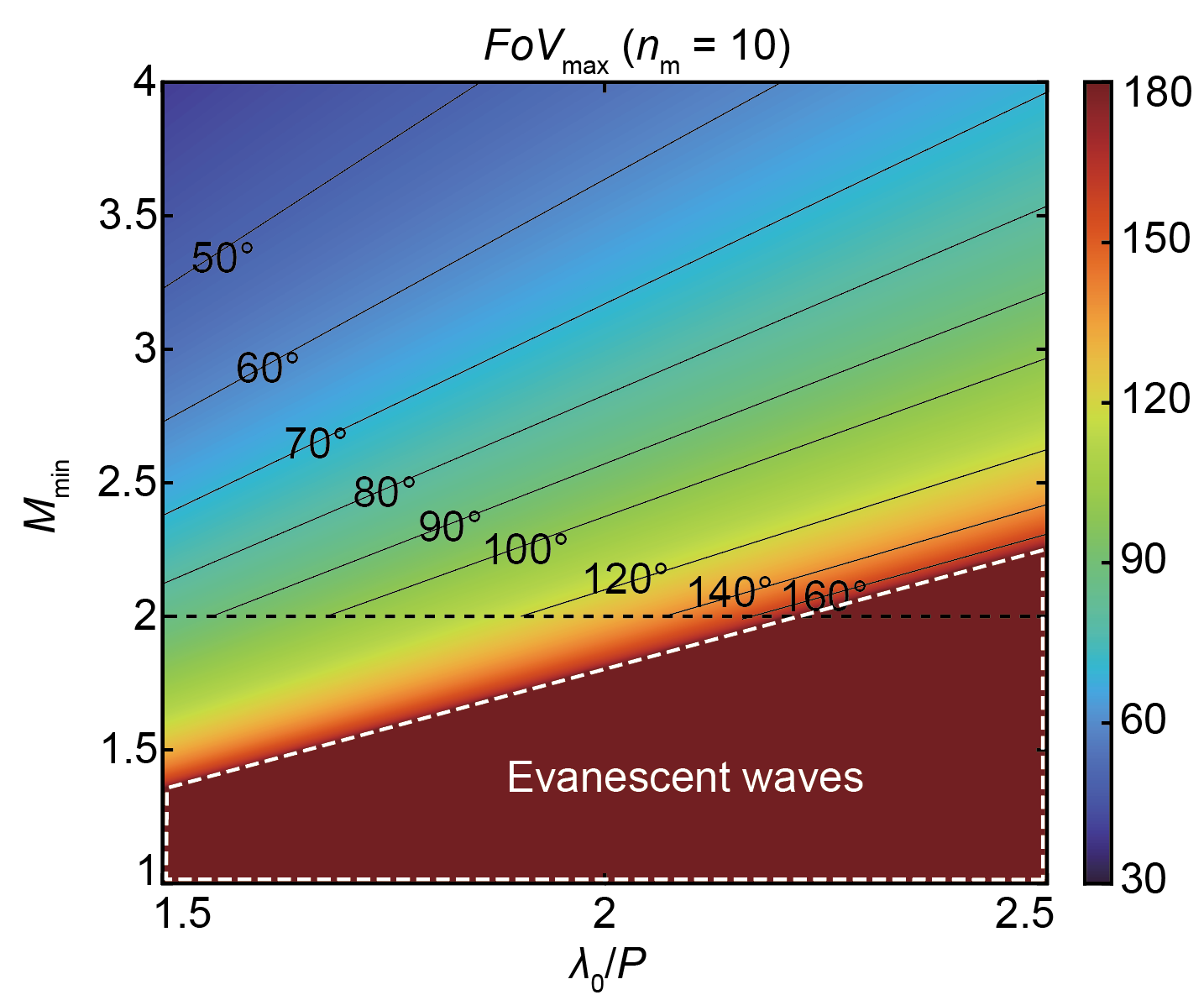


**Fig. S21** | The relationship between the maximum field of view (*FoV*_max_) and the phase sampling capability factors *λ*_0_/*P* and *M*_min_.

**Supplementary Note 19: Discussion on maximum detection distance**

To quantitatively estimate the maximum detection distance of our system, we employ the fundamental LiDAR range equation^17,18^. It is concluded that as a light beam propagates, the optical power density *ρ*_p_ (i.e., the optical power per unit cross-sectional area of the beam) is:

$$\begin{aligned} \rho_{p}=\frac{P_{t}\eta_{t}}{\pi{(\delta R)}^{2}}e^{-R\eta_{a}}\#\left( S-21 \right) \end{aligned}$$

where *P*_t_ is the optical power of the laser source, *η*_t_ is the efficiency of the optical system, *δ* denotes half-angle beam divergence in radians, and *R* denotes the detection distance. The exponential term $e^{-R\eta_{a}}$ accounts for atmospheric losses, and *η*_a_ denotes the atmospheric attenuation coefficient. Depending on the relationship between the laser spot size and the target's effective reflective area, two cases can be considered:

**Case 1: Target area larger than the beam spot size (Fig. S22a)**

$$\begin{aligned} P_{r}=\frac{P_{t}\eta_{t}\eta_{r}cos(\Omega)A_{r}}{\pi R^{2}}e^{-2R\eta_{a}}\#\left( S-22 \right) \end{aligned}$$

where *P*_r_ represents the optical power received by the detector, *η*_r_ represents diffuse reflectivity, *Ω* denotes angle of incidence, *A*_r_ denotes the receiver area illuminated by Lambertian reflection from the target illuminated area *A*_t_, assuming the metasurfaces are placed close to the receiver. Introduce the principal branch of the Lambert *W* function, *W*_0_(*x*e*^x^*)=*x* (*W*_0_>-1), and solve the equation to obtain:

$$\begin{aligned} R=\frac{1}{\eta_{a}}W_{0}\left[ \eta_{a}\sqrt{\frac{P_{t}\eta_{t}\eta_{r}\cos\left( \Omega\right)A_{r}}{\pi P_{r}}} \right]\#\left( S-23 \right) \end{aligned}$$

When air propagation attenuation is ignored (*η*_a_ = 0), we can simplify the solution to：

$$\begin{aligned} R=\sqrt{\frac{P_{t}\eta_{t}\eta_{r}cos(\Omega)A_{r}}{\pi P_{r}}}\#\left( S-24 \right) \end{aligned}$$

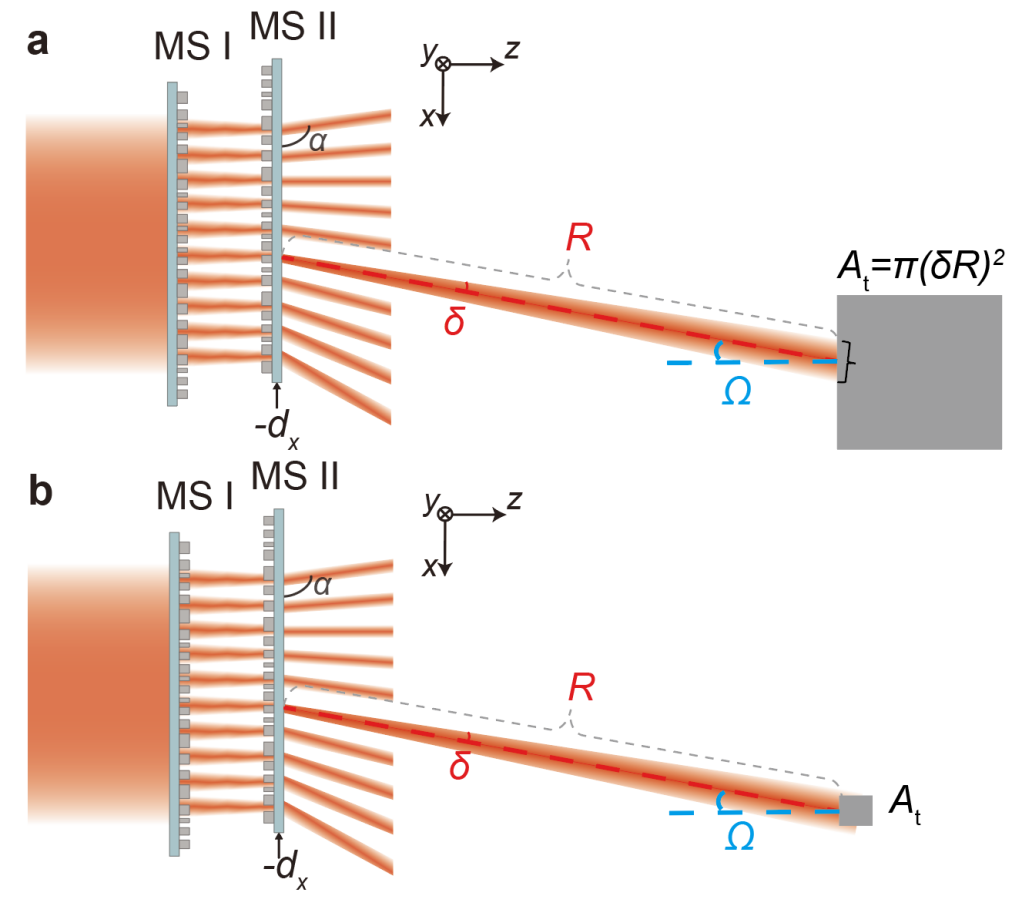


**Fig. S22**| Two cases of lidar detection beam spot and target. (a) Target area larger than the beam spot size. (b) Target area smaller than the beam spot size.

**Case 2: Target area smaller than the beam spot size (Fig. S22b)**

$$\begin{aligned} P_{r}=\frac{P_{t}\eta_{t}\eta_{r}\cos^{2} (\Omega)A_{t}A_{r}}{\pi^{2}\delta^{2}R^{4}}e^{-2R\eta_{a}}\#\left( S-25 \right) \end{aligned}$$

where *A*_t_ represents target cross-sectional area. Similarly, solve the equation to obtain:

$$\begin{aligned} R=\frac{2}{\eta_{a}}W_{0}\left[ \frac{\eta_{a}}{2}\sqrt[4]{\frac{P_{t}\eta_{t}\eta_{r}\cos^{2} (\Omega)A_{t}A_{r}}{\pi^{2}\delta^{2}P_{r}}} \right]\#\left( S-26 \right) \end{aligned}$$

When air propagation attenuation is ignored (*η*_a_ = 0), we can simplify the solution to:

$$\begin{aligned} R=\sqrt[4]{\frac{P_{t}\eta_{t}\eta_{r}\cos^{2} \left( \Omega\right)A_{t}A_{r}}{\pi^{2}\delta^{2}P_{r}}}\#\left( S-27 \right) \end{aligned}$$

Equation (S-24) shows that *P*_r_ ∝ 1/*R*² when the laser spot remains smaller than the target area, Equation (S-27) indicates *P*_r_ ∝ 1/(*θ*²*R*⁴) when the light spot larger than the target. We estimate the system’s maximum detection distance *R*_max_ based on the received optical power *P*_r_. Specifically, *R*_max_ is defined as the distance at which *P*_r_ just equals the detector’s minimum detectable power *P*_rmin_.

In our work, we achieved minimal beam divergence (0.1-0.2°) by precisely tailoring the emission profile through far-field coupled cascaded metasurfaces, designed via inverse ray tracing and validated through forward simulation. This ensures that even at relatively long distances, the laser spot is still likely to remain smaller than the target, thereby avoiding the rapid degradation in received power *P*_r_. Based on this assumption, we proceed to fit the experimental data using *P*_r_ ∝ 1/*R*² and estimate the system’s maximum detection distance. Here, we carried out measurements by placing targets at several meters and monitoring the emitted and reflected optical power from a single beam within the beam array (Fig. S23a).


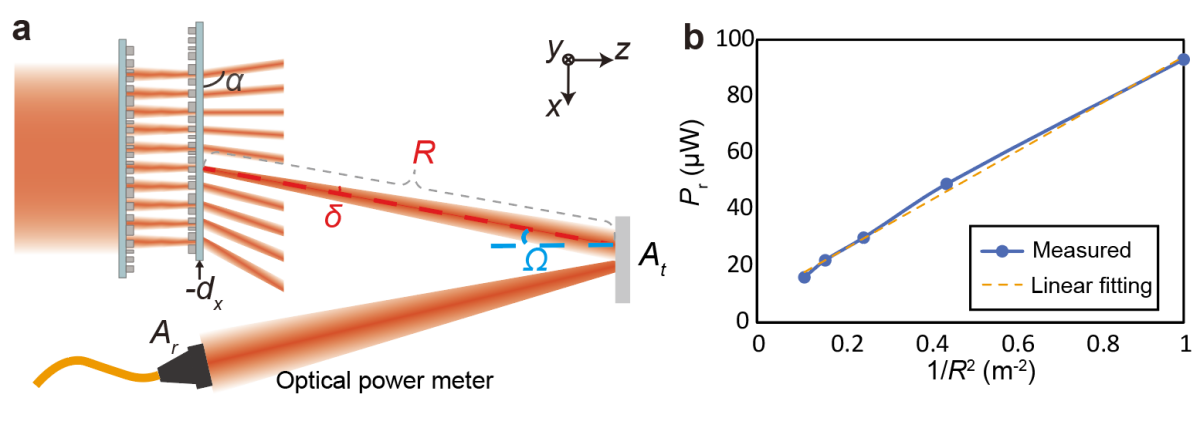


**Fig. S23** | (a) Schematic of the measured optical power *P*_r_ as a function of target distance *R* from 1 to 3 meters. (b) Linear fitting of the measured data based on the inverse square law *P*_r_ ∝ *1/R^2^*.

In the experiment, the incident optical power *P*_t_ measured on a single array unit of MS I was 143μW. The reflectivity of the target surface was approximately 80%, the incident angle was around *Ω* ≈ 10°, and the optical power meter (Daheng Optics) had a receiving area of approximately *A*_r_ ≈ 7.85×10^-3^m^2^. As shown in Fig. S23b, the received optical power *P*_r_ exhibits a linear positive correlation with the inverse square of the target distance *R*, which is consistent with the theoretical prediction. Based on the linear fitting, when the distance *R* increases to 100 m (corresponding to the typical detection range of commercial LiDAR), the calculated received power is *P*_r_ = 8.57nW, which is above the detection limit of existing commercial infrared cameras.

In practical scenarios, as the distance *R* increases, atmospheric attenuation $e^{-2R\eta_{a}}$ becomes increasingly significant, leading to a further reduction in received optical power *P*_r_. Additionally, typical targets often have low reflectivity, which exacerbates the decrease in *P*_r_. However, it's noteworthy that in our experiments, the laser illuminating a single unit of the MS I metasurface had a power of only 143 μW. To compensate for these losses and ensure sufficient detection distance, the light source power *P*_t_ and the optical efficiency *η*_t_ can be appropriately increased.

**Increasing *P*_t_**: Many commercial LiDAR systems adopt VCSEL arrays, whose arrayed emission pattern naturally corresponds to the array framework of MS I. This compatibility eliminates the need for additional beam power splitting.

**Increasing *η*_t_**: The low efficiency of current devices stems from two primary factors. First, cascaded metasurfaces presented in the manuscript suffered from low optical efficiency (~20%) due to imperfect fabrication processes, particularly electron-beam lithography (EBL) accuracy and lift-off issues. These defects caused a significant portion of the output beam to remain in the zeroth order, wasting optical energy. Through process optimization, we’ve now achieved ~60% light utilization efficiency in similar designs. Second, improvements can be made by redesigning the metasurface substrate to reduce reflection losses and suppress stray diffraction. In addition to traditional approaches such as applying anti-reflection coatings, a more integrated solution involves incorporating multilayer dielectric films directly into the substrate, enabling cascaded metasurfaces to reach transmission efficiencies as high as 86%^5^.

Overall, long-range detection can be ensured by employing high-power laser arrays, optimizing the optical efficiency of the cascaded metasurfaces, and adapting the binocular ranging system. It is worth mentioning that, for conceptual demonstrations at ranges of several hundred meters, time-of-flight (ToF) detection using a SPAD array may also be considered. The ToF-based systems offer high depth resolution and accuracy at extended distances, while lowering the minimum detectable return power, providing another system solution for long-range application based on our proposed THCMS.

**References**

1 Yu, N. F. *et al.* Light propagation with phase discontinuities: generalized laws of reflection and refraction. *Science* **334**, 333-337 (2011).

2 Ogawa, C. *et al.* Rotational varifocal moiré metalens made of single-crystal silicon meta-atoms for visible wavelengths. *Nanophotonics* **11**, 1941-1948 (2022).

3 Cai, X. D. *et al.* Dynamically controlling terahertz wavefronts with cascaded metasurfaces. *Advanced Photonics* **3**, 036003-036003 (2021).

4 Zhang, L. *et al.* Highly tunable cascaded metasurfaces for continuous two‐dimensional beam steering. *Advanced Science* **10**, 2300542 (2023).

5 He, T. *et al.* Perfect anomalous refraction metasurfaces empowered half-space optical beam scanning. *Nature Communications* **16**, 3115 (2025).

6 Chen, R. *et al.* A semisolid micromechanical beam steering system based on micrometa-lens arrays. *Nano Letters* **22**, 1595-1603 (2022).

7 Park, J. *et al.* All-solid-state spatial light modulator with independent phase and amplitude control for three-dimensional LiDAR applications. *Nature Nanotechnology* **16**, 69-76 (2021).

8 Li, S. Q. *et al.* Phase-only transmissive spatial light modulator based on tunable dielectric metasurface. *Science* **364**, 1087-1090 (2019).

9 Hsu, W.-C. *et al.* Metasurface-and PCSEL-based structured light for monocular depth perception and facial recognition. *Nano Letters* **24**, 1808-1815 (2024).

10 Wang, Q. H. *et al.* On‐chip generation of structured light based on metasurface optoelectronic integration. *Laser & Photonics Reviews* **15**, 2000385 (2021).

11 Kim, G. *et al.* Metasurface-driven full-space structured light for three-dimensional imaging. *Nature Communications* **13**, 5920 (2022).

12 Ni, Y. *et al.* Metasurface for structured light projection over 120 degrees field of view. *Nano Letters* **20**, 6719-6724 (2020).

13 Jing, X. *et al.* Single-shot 3D imaging with point cloud projection based on metadevice. *Nature Communications* **13**, 7842 (2022).

14 Rubin, N. A., Shi, Z. & Capasso, F. Polarization in diffractive optics and metasurfaces. *Advances in Optics and Photonics* **13**, 836-970 (2022).

15 Simpson, G. R. in *1981 IEEE MTT-S International Microwave Symposium Digest.* 507-509 (IEEE).

16 Prod'homme, H. & del Hougne, P. Updatable Closed-Form Evaluation of Arbitrarily Complex Multi-Port Network Connections. *arXiv preprint arXiv:2412.17884* (2024).

17 Jenn, D. *Radar and laser cross section engineering*. (American Institute of Aeronautics and Astronautics, Inc., 2005).

18 Williams, G. & Huntington, A. LIDAR effective range. *Allegro Microsyst* (2023).
